# Retrospective analysis of factors influencing the implementation of a program to address unprofessional behaviour and improve culture in Australian hospitals

**DOI:** 10.1186/s12913-023-09614-1

**Published:** 2023-06-07

**Authors:** Kate Churruca, Johanna Westbrook, Kathleen L Bagot, Ryan D McMullan, Rachel Urwin, Neil Cunningham, Rebecca Mitchell, Peter Hibbert, Neroli Sunderland, Erwin Loh, Natalie Taylor

**Affiliations:** 1 Australian Institute of Health Innovation, Macquarie University, Level 6, 75 Talavera Rd, Macquarie Park, 2109 NSW, Australia; 2 Nursing Research Institute, St Vincent’s Health Australia Sydney, St Vincent’s Hospital Melbourne and Australian Catholic University, Daniel Mannix Building, Brunswick Street, Fitzroy, Australia; 3 St Vincent’s Hospital Melbourne, Fitzroy, Australia; 4 Allied Health and Human Performance, University of South Australia, Adelaide, Australia; 5 St Vincent’s Health Australia, Melbourne, Australia; 6 School of Population Health, University of New South Wales, Sydney, Australia

**Keywords:** Unprofessional behaviour, Professional accountability program, Culture change, Hospital culture, CFIR, Implementation determinants, Evaluation

## Abstract

**Background:**

Unprofessional behaviour among hospital staff is common. Such behaviour negatively impacts on staff wellbeing and patient outcomes. Professional accountability programs collect information about unprofessional staff behaviour from colleagues or patients, providing this as informal feedback to raise awareness, promote reflection, and change behaviour. Despite increased adoption, studies have not assessed the implementation of these programs utilising implementation theory. This study aims to (1) identify factors influencing the implementation of a whole-of-hospital professional accountability and culture change program, *Ethos*, implemented in eight hospitals within a large healthcare provider group, and (2) examine whether expert recommended implementation strategies were intuitively used during implementation, and the degree to which they were operationalised to address identified barriers.

**Method:**

Data relating to implementation of *Ethos* from organisational documents, interviews with senior and middle management, and surveys of hospital staff and peer messengers were obtained and coded in NVivo using the Consolidated Framework for Implementation Research (CFIR). Implementation strategies to address identified barriers were generated using Expert Recommendations for Implementing Change (ERIC) strategies and used in a second round of targeted coding, then assessed for degree of alignment to contextual barriers.

**Results:**

Four enablers, seven barriers, and three mixed factors were found, including perceived limitations in the confidential nature of the online messaging tool (‘Design quality and packaging’), which had downstream challenges for the capacity to provide feedback about utilisation of *Ethos* (‘Goals and Feedback’, ‘Access to Knowledge and Information’). Fourteen recommended implementation strategies were used, however, only four of these were operationalised to completely address contextual barriers.

**Conclusion:**

Aspects of the inner setting (e.g., ‘Leadership Engagement’, ‘Tension for Change’) had the greatest influence on implementation and should be considered prior to the implementation of future professional accountability programs. Theory can improve understanding of factors affecting implementation, and support strategies to address them.

**Supplementary Information:**

The online version contains supplementary material available at 10.1186/s12913-023-09614-1.

## Background

Unprofessional behaviour, encompassing the overtly aggressive (e.g., shouting) to the uncivil or passively hostile, [1-3] is increasingly recognised as a problem in healthcare; a recent survey of seven Australian hospitals found close to 40% of staff experienced at least weekly incivility or bullying from co-workers [4]. Unprofessional behaviour negatively impacts on teamwork, communication, technical performance and clinical decision-making, having detrimental effects on patient care [5-11] and employee wellbeing [12, 13].

Programs have been developed to address unprofessional behaviour in hospitals, with the Promoting Professional Accountability Program by Vanderbilt University Medical Center the most prominent example [2,18,14-]. The program includes supportive policies, surveillance tools to capture reports of unprofessional behaviour by physicians, and a tiered model of intervention which begins with informal, nonpunitive peer feedback for less severe behaviour (e.g., a cup of coffee conversation with a trained peer) and escalates in formality for severe or persistent behaviour, which could ultimately result in disciplinary action (e.g., removal of hospital privileges). The aim of the program is to encourage self-reflection and course correction among physicians engaging in unprofessional behaviour and challenge the normalisation of such behaviour [2, 15].

The Vanderbilt program has shown success in lowering the risk profile of physicians in terms of patient complaints, [16] as well as sustaining high levels of hand hygiene adherence when used as part of a wider behaviour change initiative [17]. Elements have been taken up as part of patient safety initiatives elsewhere, [19, 20] including in Australia [21]. Research on implementation has been limited, with one study by McKenzie, Shaw [21] exploring factors influencing the implementation of a multicomponent accountability program at a large Melbourne hospital. It found that the implementation climate was an enhancing factor, while compatibility with working conditions was a limiting factor. Leadership commitment was simultaneously an enabler and a barrier because while leader support was crucial to driving implementation, some leaders did not consistently model professionalism. Likewise, aspects of the implementation process were both an enabler and a barrier as communications about the program developed staff awareness, but there was inadequate reinforcement of key messages. No study has systematically investigated determinants of implementing professional accountability programs using implementation theory.

### Using theory to understand and address factors affecting implementation

**Fig. 1:**
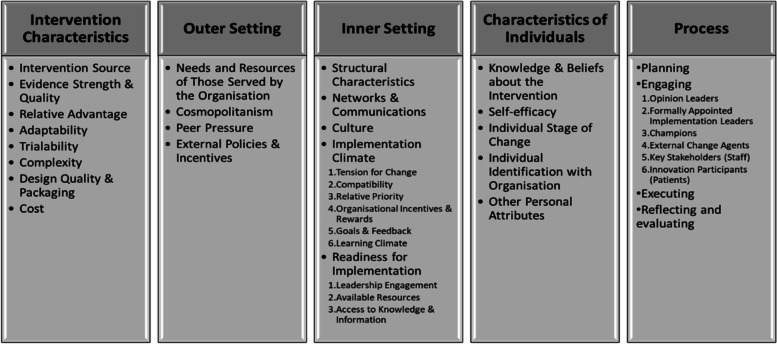
CFIR domains, constructs and subconstructs [22]. *Box=domains; dot points=w (sub)constructs within domain*

The Consolidated Framework for Implementation Research (CFIR) [22] is a typology of the determinants of implementation success consisting of five domains with constructs and subconstructs (Fig. 1). Application of this framework supports an understanding of factors affecting implementation in a particular context, as well as enabling comparison and the ability to generalise these findings to other settings [23].

Implementation strategies have been compiled and matched to the CFIR’s constructs through a consensus-based approach: the Expert Recommendations for Implementing Change (ERIC) [24, 25]. Using the CFIR to categorise implementation determinants then facilitates the development of strategies to address barriers by applying the ERIC matching tool. For example, if, during implementation, the processes of a new program are found to have poor integration with existing workflow, Compatibility would be categorised as an implementation barrier. One ERIC-matched strategy for this subconstruct is to conduct local consensus discussions to better understand staff needs and existing processes [25].

However, frontline implementers may lack access to knowledge, expertise, time, or resources to utilise implementation theory optimally during the introduction of multi-faceted programs [26, 27]. In such cases, retrospective application of theory is one way of advancing the science of implementation and informing the design of evidence-based approaches in the future [28, 29]. For example, ERIC can be used to address identified barriers by selecting future implementation strategies [29]. Alternatively, where implementation strategies have already been used intuitively by implementers, we can evaluate the degree to which they are theoretically supported and appropriately tailored to matched barriers. Such analysis (a) provides insights into implementers’ tacit knowledge and capability in identifying implementation challenges and designing appropriate approaches to overcome them, and (b) contributes to the standardisation, replicability and generalisability of the implementation of these programs in other contexts [30]. A retrospective analysis of determinants and implementation strategies can also showcase where theory may be less relevant to guide implementation, and where it may be most useful (e.g., in addressing more complex challenges), thereby making the implementation process more efficient [30].

### The present study

A professional accountability and culture change program, *Ethos*, was implemented in eight hospitals across Australia, providing opportunity to examine implementation determinants for such programs at scale. The aims of this study were to retrospectively:

1. Identify key factors influencing the implementation of the *Ethos* program; and.
2. Examine whether expert recommended implementation strategies were intuitively used during implementation, and, where identified, the degree to which they aligned with barriers.

## Method

**Fig. 2:**
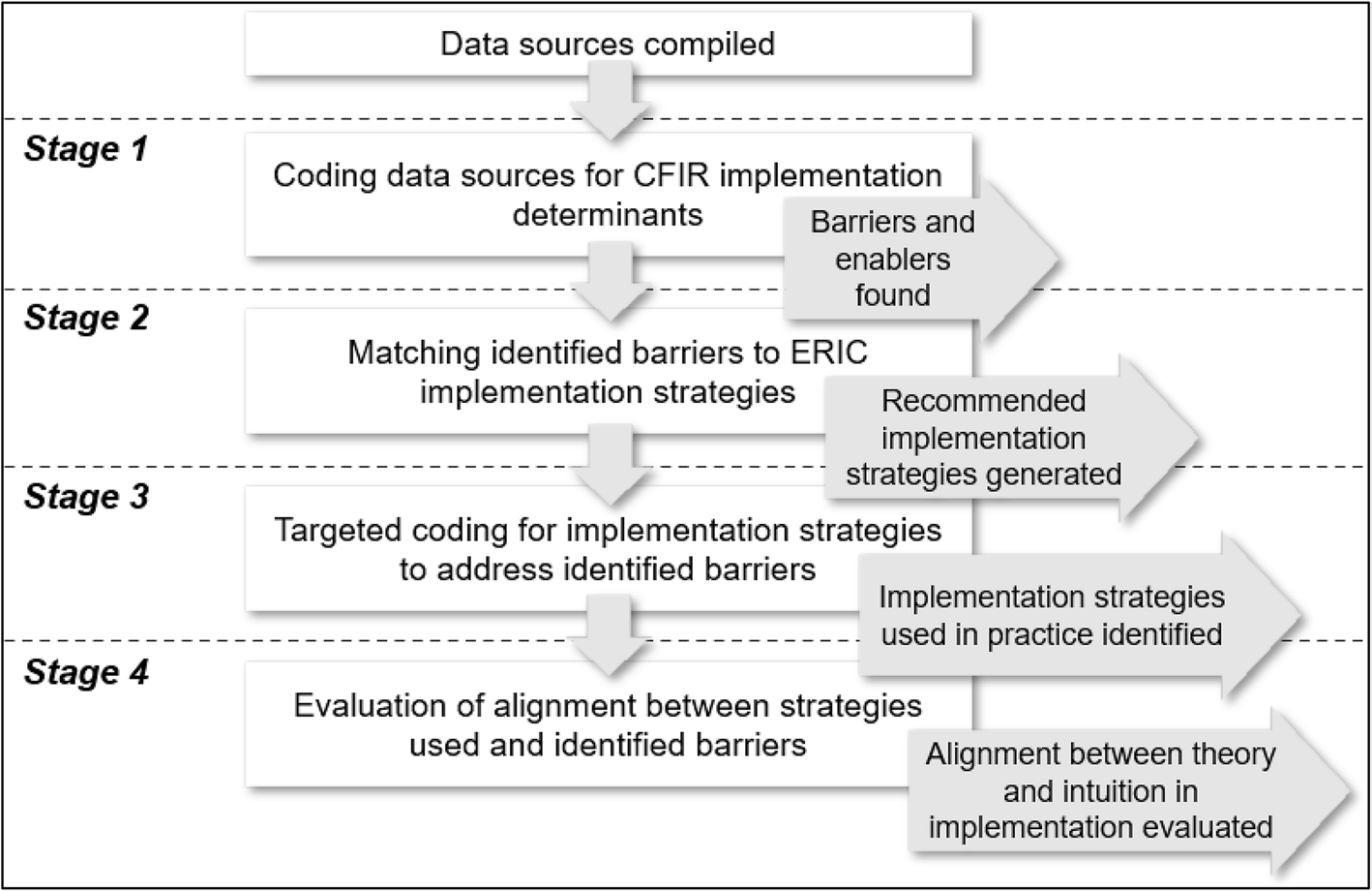
Schematic of method for identifying implementation determinants and strategies to address barriers

This study, part of a larger program of research to evaluate the *Ethos* program, drew upon organisational documents and primary empirical data. Data were coded according to the CFIR, [22, 23] and a list of implementation strategies to address identified barriers was generated using ERIC [24, 25]. Where a strategy was used, its operationalisation by program implementers was evaluated for the degree to which it aligned to its matched barrier in context. The full analysis process is depicted in Fig. 2. Study reporting follows the Template for Intervention Description and Replication (TIDieR; Supplementary File 1) [31] and the Standards for Reporting Qualitative Research (SRQR; Supplementary File 2) [32].

### The intervention

The aim of *Ethos* is to change culture by addressing unprofessional behaviour *and* recognising behaviours demonstrating professionalism. It is a whole-of-hospital program where both clinical and nonclinical staff are empowered to speak up against unprofessional behaviour in the moment. If they are unable to do so they can provide feedback about this behaviour (Feedback for Reflection) or positive behaviours promoting safety and quality (Feedback for Recognition) using an online messaging system with the option of staying anonymous. Submissions are triaged by a trained multidisciplinary team and then sent on to either a peer messenger or line manager who delivers the message. Further details on *Ethos* are provided elsewhere [33].

### Implementation

*Ethos* was implemented in a staged way across eight hospital sites in an organisational network (the Group), beginning in mid-2017, with the last site receiving the program in February 2020. Hospitals included public and private, and spanned three Australian states. The online messaging infrastructure was established at Group-level, with a steering committee responsible for engaging and training the hospital-level executive team. Following this, a “train-the-trainer” model was used to support mandatory training for all staff. After the program had been implemented at two early-adopter sites, an independent review was conducted, and this led to changes to some program language; for example, to reduce formality, “reporting” was amended to “messaging”.

### Data sources

All qualitative data generated during the research program, in addition to organisational documents about *Ethos* provided by the Group, were used in the analysis. Primary data included interviews with middle and senior managers with varying levels of formal involvement in *Ethos*, and surveys of staff working at the hospitals. Organisational documents provided complementary naturalistic data on the program. Data were generated or collected at varying time points and not all sites contributed to all sources (see Table 1). Data sources also varied in terms of content due to the perspective(s) captured within the data source. These differences are considered in the Results, where discrepancies between sources and stakeholders are highlighted. Data sources were incorporated into the analysis until data saturation was reached, with only a few complementary and no novel insights gleaned from data from the mid-to-late stage of implementation.

**Table 1:** Sources of data and their role in analysis of implementation of *Ethos*

Data source	Implementation stage generated or collected	Details
*Organisational documents*		
1. Program information and implementation plan	Early (2017)	11-page document developed by the Group that provides background on the program, the need for it, the components of *Ethos*, and high-level plans for its implementation. ***Analysis***: Coded to the CFIR.
2. Independent review of *Ethos* program in two hospitals	Early to mid (March - July 2019)	14-page report of an independent review into *Ethos* at two sites first to implement (one public, one private). Review was commissioned by the Group *Ethos* steering committee and aimed to identify elements working well and areas for improvement. ***Analysis***: Coded to the CFIR.
***Empirical data collection***		
3. Baseline survey of all staff [4]	Early (December 2017 – November 2018)	***Sampling***: All staff from seven hospitals prior to implementing Ethos were invited to undertake a survey about their experiences of unprofessional behaviours. Response rate was 34% (*n* = 5,178). ***Collection***: Included closed and open response questions on experience of unprofessional behaviour in the preceding 12 months. Further details on the survey are provided elsewhere [4, 13]. ***Analysis***: Only open responses to questions about unprofessional behaviour and behaviour in general within the hospital were analysed. 32% of respondents (*n* = 1,636) answered at least one of these open questions. Responses coded to the CFIR.
4. Interviews with senior hospital leadership	Early to mid (November 2018)	***Sampling***: Interviews (*n* = 15) with all consenting executives from the two sites (one public, one private) that were first to implement Ethos. ***Collection***: Interview schedule covered views on unprofessional behaviour in the hospitals and the *Ethos program along with* their perspectives on the results of the baseline survey results. ***Analysis***: Transcribed and coded to the CFIR.
5. Survey of Ethos peer messengers	Mid (October – November 2020)	***Sampling***: All current and previous *Ethos* peer messengers from eight sites invited to take part. There were 60 respondents (response rate 41.5%). ***Collection***: Survey asked open and closed response questions about reasons for taking on the role, experiences of the program, training, support, challenges. Further details on the survey are provided elsewhere [34]. ***Analysis***: Open responses coded to the CFIR.
6. Interviews with middle managers	Mid to late (August 2020 – May 2021)	***Sampling***: Purposive sampling of *n* = 30 middle managers from five sites to explore unprofessional behaviours and the *Ethos* program. Participants were clinical (nursing = 12, medicine = 8) and support services (*n* = 10) and some had formal roles within *Ethos* (e.g., trainers). ***Collection***: Interview schedule covered descriptions of unprofessional behaviour experienced and raising by staff and how middle managers responded to these concerns, and views about the Ethos program. ***Analysis***: Interview recordings were transcribed and inductively coded; code on “implementation” was extracted for this analysis and recoded to the CFIR.

### Data analysis

#### Stage 1. Identification of implementation determinants

All qualitative data were imported into NVivo and coded deductively according to the CFIR’s five domains and 39 constructs/subconstructs. During coding, data were read through to identify passages of text that met the inclusion criteria for a CFIR construct (e.g., *“I believe all staff should be empowered to have the confidence to report bad behaviour and I think this is a great initiative if implemented”* was coded to the ‘Knowledge & Beliefs’ construct of the ‘Characteristics of Individuals’ domain). Coding was conducted by KC (PhD, experienced health services and implementation researcher) and reviewed by NT (PhD, behaviour change and implementation specialist).

A synthesis and rating process was then undertaken, informed by the CFIR guide on coding qualitative data [35]. KC read all coded data within a construct, wrote a summary of the content with reference to the sources and stakeholders represented, and whether the coded data was negative or positive, and would therefore hinder or facilitate implementation. Based on this, it was classified as an enabler, barrier, or mixed determinant on the implementation process; mixed ratings were only used where there was a high degree of inconsistency between sources and/or stakeholders. The frequency with which a construct was present in the data was also considered and rated as not present, minimal, moderate, or high presence, depending on both the number of data sources and prevalence within those sources. Only CFIR (sub)constructs with high presence in the data were deemed sufficiently salient to consider implementation determinants.

#### Stage 2. Matching barriers to ERIC implementation strategies

The CFIR-ERIC implementation strategy matching tool [36] was utilised to identify all relevant implementation strategies to address barriers found. The tool is a macro-enabled Excel file developed based on research with implementation experts in which CFIR barriers were matched to a list of implementation strategies [25]; a barrier is matched to a strategy with either majority endorsement (Level 1 = > 50% of experts agreed the strategy would address that (sub)construct) or a top quartile endorsement (Level 2 = 20–49% of experts agreed). For example, a Level 1 strategy in the inner setting barrier of culture would be to “identify and prepare champions” (52%), while a Level 2 strategy would be to “inform local opinion leaders” (22%).

In the matching tool, it is possible to match individual constructs to strategies, but also to input all identified barriers simultaneously as a query and generate a list of strategies endorsed for addressing the range of barriers in one’s setting. A cumulative percentage is provided for each strategy to demonstrate its aggregate degree of expert endorsement for addressing the configuration of barriers in the query. We used a query in the present study to calculate a cumulative percentage and strategies with a cumulative endorsement of > 50% for the range of identified barriers were considered.

#### Stage 3. Targeted coding of implementation strategies

We used these strategies and their descriptions [24] to conduct a second round of targeted coding of data represented in Table 1. In addition, we reviewed all other information on implementation compiled from notes by the research team during attendance and observation of various meetings (e.g., research project steering committee, *Ethos* working group). For each ERIC-recommended implementation strategy identified as represented, we developed a description of how it was operationalised by *Ethos* implementers.

Stage 4. Alignment between strategies used and identified barriers.

To account for each barrier’s specific contextual factors, an assessment was made by KC and NT of the degree (scale 0 to 2) to which the implementation strategy as operationalised addressed its matched barrier(s). A rating of (2) aligned to contextual barrier was given when the strategy fully addressed the barrier described, (1) partially aligned when the strategy addressed parts of the barrier, or not aligned (0) when the strategy did not address the barrier as described. Where multiple barriers were addressed by a single strategy, alignment was evaluated for each barrier individually and then an overall assessment was calculated by averaging ratings.

## Results

Fourteen CFIR constructs were represented by the coded data and found to influence the implementation of the *Ethos* professional accountability program: seven barriers, four enablers, and three for which evidence was mixed (i.e., data among stakeholders and sources were balanced between positive and negative). A summary of each determinant is presented in Table 2 and described with supporting quotes below, with the full analysis and data in Supplementary File 3.

**Table 2:** Determinants affecting implementation success for a hospital professional accountability program

Type of determinant	Domain	Construct	Summary of determinant in the context of the Ethos program implementation
Barrier	Intervention characteristics	Design Quality & Packaging	Confusion about both ‘positive’ and ‘negative’ feedback in the same messaging tool, the labelling of feedback in this way, concerns about anonymity and a lack of ‘natural justice’.
	Inner setting	Networks & Communications	Poor communication vertically and horizontally, despite procedures in place for consultation. Communication characterised by lack of transparency, hierarchy, and tribalism.
		Implementation climate - Goals & Feedback	Broad goals but lack of clarity about what the program would achieve, perpetuated by the dearth of feedback at both an individual and organisational level on usage of *Ethos*.
		Implementation climate - Learning Climate	Hospital settings, leadership and climate were viewed as frequently hostile and punitive when issues were raised. Spurious focus on quality improvement.
		Readiness for implementation - Leadership Engagement	Staff perceived leaders as not accountable with a poor track record of addressing unprofessional behaviours. Senior leaders were supportive of *Ethos* but were not core implementers and were not always knowledgeable on the program.
		Readiness for implementation - Access to Knowledge & Information	Training not accessible to all, visibility of *Ethos* declined over time and specific information on the messaging process was inadequate.
	Characteristics of individuals	Knowledge & Beliefs about the Intervention	Many were distrusting, sceptical or cautious in their view of *Ethos* and its potential effectiveness.
Enabler	Inner setting	Structural Characteristics	On balance, sense that the Group was values-based, which positively impacted how staff interacted with one another.
		Implementation climate – Tension for Change	Unprofessional behaviour was widely considered a problem, though some expected it would continue to be tolerated by leadership.
	Process	Engaging - Champions	*Ethos* peer messengers volunteered or were invited to take on the role and were intrinsically motivated by the work.
		Reflecting & Evaluating	Clear commitment to, and evidence of, evaluation and utilising those insights to revise and update *Ethos* and its implementation.
Mixed	Inner setting	Culture	Inconsistent views of the culture as respectful and driven by benevolent values, versus hostile, unequal and driven by a financial imperative.
		Implementation Climate –Compatibility	Delivery of feedback and respectful interaction pillars of professional conduct; limited integration of the program with other systems, particularly HR.
		Implementation Climate – Relative Priority	Differential perspectives related to stakeholder group; middle managers and most staff viewed the program as low priority, whereas peer messengers saw it as important.

### Barriers to *Ethos* implementation

*Design Quality and Packaging* was a barrier with many staff sceptical that feedback provided in the online messaging tool could be anonymous, particularly as submissions required a high level of detail; the person being referred might be able to discern the situation and referrer, raising concerns about reprisal. For example, one staff member commented:

*The Ethos idea is flawed. I’d like to make some comments about specific staff, but … you need specific examples... This completely unblinds the anonymity of the process! I am afraid of being victimised… (Baseline survey respondent H1.ID64)*

Many participants also viewed the feedback messaging process as unfairly one-sided, because the referred person had no “right of reply”. The packaging of *Ethos* was further criticised as being more formal than intended and weighted toward the Feedback for Reflection (perceived negative feedback). Indeed, some said the inclusion of positive and negative submissions within the same system was inappropriate, questioning why positive feedback could not just be provided publicly or in situ.

*Networks and Communication* was a barrier from the Inner Setting, with numerous participants across sites reporting poor vertical communication with leadership, between professional groups and departments, and between clinical staff and non-clinical management. While some mentioned processes for communication and consultation, they suggested these were not always adhered to. These comments, below, highlighted a lack of transparency, rigid hierarchy and tribalism affecting the flow of information and social networks.

*There is a consistent culture of withholding information from clinical staff (despite monthly updates which are generally informationless) and of asking clinical staff for input which is then ignored. The opacity with which the hospital is run makes angry, anxiety-driven behaviour of clinical staff more likely. (Baseline survey respondent H3.ID46)*

*There remains a hierarchy/tribe mentality with doctors/nurses/allied health/other groups treated differently by the organisation depending on their status. This reinforces behaviour with staff. (Baseline survey respondent H1.ID149)*

*Goals and Feedback*, part of the Implementation Climate, was another barrier, with stakeholders suggesting that while general organisational goals were communicated, their monitoring, feedback, and performance management were inadequate. Specifically for *Ethos*, broad goals were articulated in the program information and implementation plan. Nevertheless, some stakeholders said that the program’s purpose was not well communicated or made relevant to all staff. Feedback was also deemed inadequate. At an individual level, the confidentiality of the messaging system precluded those who made an *Ethos* submission getting information about how a message had been received:

*I have already put in an Ethos negative report about treatment to me from a staff member but have received no acknowledgement or feedback to date. (Baseline survey respondent, H1.ID87)*

At an organisational level, also in part due to confidentiality, there was little to no reporting of data on the utilisation and outcomes of *Ethos* to staff:

*… we need to close the feedback loop… I don’t know the stats. Is it about one third positive, two thirds negative, rough figures?... Maybe there’s opportunities to sort of harness them a bit more but understand it’s confidential… How do we feed back to staff about what is being addressed? What are some of the negative things being addressed? And you can’t obviously make anything identifiable. (Senior hospital leader 180)*

This further contributed to confusion about what *Ethos* was achieving, with the lack of information on the breakdown of positive versus negative feedback giving the impression that most *Ethos* submissions were about negative behaviour.

*Learning Climate* was another barrier of the Implementation Climate. Numerous baseline survey respondents mentioned their workplace was hostile, punitive, or ineffective in dealing with safety and clinical issues, and that there was “*a culture of blame and finger pointing if any errors occur*” (Baseline survey respondent, H2.ID36). Some reported that the focus on evidence-based practice and quality improvement were limited and that many staff did not feel able to speak up. While some senior hospital leaders conceded there were weaknesses in the learning climate, they mentioned being in a process of transitioning their hospital to a greater focus on improvement, with one saying:

*We’ve taken a journey approach to how we embed improvement thinking… it started two and a half years ago. (Senior hospital leader 140)*

Two subconstructs under Readiness for Implementation were barriers. First, in terms of *Leadership Engagement*, baseline survey responses indicated generally poor accountability among leadership, including lack of transparency; that management had not addressed bullying or unprofessional behaviour in the past; and were disconnected from the complexities of frontline work. While, interviewed senior hospital leaders were broadly positive about *Ethos*, they varied in their knowledge of the program and how it was being implemented, or were uncertain about their role in supporting it:

*… the key thing for us now is what are our actions. As an exec team and other leaders… I don’t think you can have one specific action I think it’s going to have to be, I think around engagement. And within that, behaviours, around leadership. (Senior hospital leader 130)*

A couple also mentioned only being in their role for a short time, or that they were soon to be leaving the hospital to move on to other opportunities.

The information and implementation plan included a multipronged strategy for communicating about *Ethos*. Nevertheless, numerous stakeholders suggested there was insufficient *Access to Knowledge and Information*, with training sessions hard for all staff to attend (e.g., scheduled on a day one did not work). Promotion materials were also inadequate, with comments on the decreased visibility of *Ethos* over time and that contributing to limited awareness:

*I think people have forgotten about the program, and don’t readily use it. (Peer messenger survey respondent, ID35).*

There were also mentions of specific knowledge gaps related to what types of behaviour were able to be reported and how negative messages were triaged, and feedback delivered. Furthermore, comments from some staff pointed to a misunderstanding of the program and how it was supposed to work. While most of these comments suggested impediments to the sustained use of the program over time, a couple of stakeholders were of the view that information provided was sufficient and there was good general knowledge among their staff.

*Knowledge and Beliefs about the Intervention* was overall rated as a barrier, though perspectives varied. Among staff responding to the baseline survey, beliefs about *Ethos* spanned negative to positive. The more numerous negative comments covered a dislike of how the program worked, lack of belief in its effectiveness, distrust in the process, and concerns that it might be ill-used or cause more harm than good:

*The Ethos program is counterproductive. It is akin to the Stasi whereby individuals can report others secretly and with no evidence of wrongdoing. It is lazy of the hospital to implement this programme without proper protocols for unwanted behaviour. (Baseline survey respondent, H1.ID175)*

Less frequent positive responses articulated belief in *Ethos*’ validity, effectiveness, and suitability for addressing the problem of unprofessional behaviour. Other sources indicated broad support for the goals of the program but were circumspect about how effective it would be, suggesting *Ethos* was not a ‘silver bullet’ to address cultural issues and could have unintended consequences:

*…it’s probably not perfect, and that doesn’t necessarily get all the right discussion and everything on the, on the table… it should provide enough warning signs... it won’t disappear quickly but as the evidence would be, yeah. And I guess the danger, the danger could be, and I have, this is, absolutely no knowledge but you hope, you’d hope some of this, it doesn’t drive it underground too. (Senior hospital leader 150).*

Medical staff were particularly vocal in negative opinions of the program, with some suggesting that it unfairly targeted them.

### Enablers to *Ethos* implementation

*Structural Characteristics* was an enabling aspect of the Inner Setting. Overarchingly, participants drew attention to the value-based nature of the wider organisational Group, often highlighting how it had developed from a charitable organisation with a Catholic ethos. Some suggested that this contributed to a positive working environment, made the organisation a desirable place to work, and aligned with the principles of the program. The following extract demonstrates this:

*I feel the culture encourages respect. I like the emphasis on values in my workplace left by the Nuns who began the organisation. (Baseline survey respondent, H5.ID70)*

However, there was some variability between hospitals on structural characteristics (e.g., public or private, large or small) which seemed to influence organisational goals (e.g., focus on money making), bureaucracy, and how inclusive the hospital was of new staff.

A subconstruct under the Implementation Climate construct, *Tension for Change* was an enabler, with nearly all sources and stakeholders recognising the problem of unprofessional behaviour that *Ethos* was designed to address. However, some suggested that those in senior positions had frequently tolerated such behaviours in the past and were sceptical about whether *Ethos* could make a difference:

*I wonder how badly a staff member would have to behave towards others to actually lose their employment. I have seen some particular staff members behave in a bullying manner for many years with no apparent ramifications. (Baseline survey respondent, H6.ID119).*

That is, while individually staff implied that the situation was intolerable, many expected that within their hospital it would continue to be tolerated by senior management. Senior hospital leadership conceded that there had been acceptance of poor behaviour for a long time, but some indicated that this was changing, and the implementation of *Ethos* reflected that.

Under the Engaging construct of the Process domain, peer messengers were identified as *Champions* of the program, because they took on a challenging and visible role to support *Ethos*. These individuals represented various clinical and non-clinical professions, and in terms of engagement strategies, many volunteered. They largely had altruistic motivations, believing in the program’s goals, and wanting to improve their organisation, although some also recognised professional development opportunities. A few were also sought out by leadership because of their professional standing within their hospital:

*I thought it would be a great chance to learn and help develop how to deal with situations that arise in the workplace. (Peer messenger survey respondent, ID17)*

…*for the Ethos program you have doctor involvement, so VMOs [visiting medical officers] who become messengers. What I like to say about the [hospital name] is we didn't have to ask any of the doctors... we had two doctors who are employed by us that were part of the program, but the other four came forward… I would say that one of them in particular must have had a pretty rough time in their training days... (Middle management, H4.ID1)*

In terms of ongoing engagement, many valued the debriefs and workshops set up among peer messengers but suggested these did not occur frequently enough. There were challenges in the role, not only due to the confronting interpersonal dynamics, but also the administrative and time burdens, and no formal incentive was provided. Some mentioned finding reward in doing their job well, helping co-workers to recognise and improve their behaviour.

Finally, *Reflecting and Evaluating* was an enabler because there was a clear commitment by the Group and influential stakeholders to this part of the process. For example, an independent review was commissioned to understand how the program was working in the two sites that were first to implement; this led to recommended revisions, many of which were enacted by the Group. *Ethos* was also being evaluated through a partnership with an academic institution, of which this paper forms one output. Senior hospital leaders spoke about the importance of evaluation, though this was mostly concerned with how to demonstrate program effectiveness, rather than implementation outcomes. However, some mentioned wanting insight into whether (adoption) and how (fidelity) *Ethos* was being used, as well as indicators of penetration and acceptability:

*… what I will be very interested in seeing at the end… how many Ethos reports are made, how many of them are unactionable. And how many of those are getting to the, the end of the behaviour that we’re actually trying to address because, you know, is it being used, I guess in a way that isn’t helpful. The other thing I’ll be very interested in obviously, the outcomes. So, what are the experiences of people who have been delivered an Ethos message and where did it, where did it feel right and where didn’t it feel right. You know, I guess my other concern is how skilled our triage team. (Senior hospital leader 170)*

### Mixed determinants of *Ethos* implementation

There were conflicting views of the *Culture* across the Group, within each hospital and even between departments. Stakeholders from some hospitals indicated there was a culture of division, bullying, favouritism, blame and fear, while others within the same hospital suggested it was a positive “inclusive, respectful, and extremely pleasant” place to work. Sometimes stakeholders mentioned a business or financial focus as driving their hospital, while others highlighted the benevolent values that had historically shaped the organisation, indicating they were still important in guiding staff behaviour:

*There is loads of advertising about our hospital values etc. They have absolutely zero of them towards staff if it saves them even a single dollar. (Baseline survey respondent, H1.ID4)*

There were also differential views about the extent of *Compatibility* of Ethos with the systems and values of the implementing hospitals. Some suggested that the principles and processes of *Ethos* were complementary to existing systems of organisational, management and professional development. On the other hand, several stakeholders were uncertain about the integration of *Ethos* with existing human resource (HR) processes and whether the balance between formal and informal action was appropriate:

*I don’t think HR are equipped to manage behavioural problems with staff… so I am unsure of how escalating problems through Ethos will eventuate in any kind of outcome if it goes to HR. (Baseline survey respondent, H1.ID286).*

A senior hospital leader spoke about the difficulty in “marrying” insights from *Ethos* data with other hospital data, such as from HR, which curtailed the ability to generate useful insights about workplace culture issues.

There were also mixed impressions about the *Relative Priority* of *Ethos*. Some staff participating in the baseline survey indicated there were other issues that needed to be attended to by their hospital, before or instead of *Ethos*. For example:

*Whilst I think this is a worthy program… I have not experienced many specific ‘unpleasant behaviours’, I do think that overall improved communication within the hospital would make an enormous difference in terms of efficiency and patient outcomes. (Baseline survey respondent, H1.ID236).*

However, many peer messengers were very positive about the program; they believed in its principles and had become involved formally to ensure that it was a success. Middle managers, interviewed late in the implementation process, reported that there was not a sustained interest in *Ethos*, that staff were occupied with direct patient care and did not see the required training as a priority.

### Implementation strategies used to address barriers

The CFIR-ERIC matching process identified 35 implementation strategies with a cumulative endorsement > 50% that were therefore recommended to address the range of barriers reported in the previous section. We found no evidence of use for eighteen strategies (e.g., inform local opinion leaders, alter incentives/allowance structures) during the implementation of *Ethos*. A further three were deemed not applicable because they were patient-focused strategies for clinical innovations (e.g., increase demand).

Fourteen generic implementation strategies recommended by ERIC were used during the implementation of *Ethos*, amounting to more than a third of expert endorsed strategies being utilised to some degree by implementation leaders. Where a strategy matched to multiple CFIR constructs (e.g., ‘conduct educational meetings’ was endorsed for four barriers), we evaluated alignment of strategy individually for each barrier, and then made an overall assessment. Overall, four of the implementation strategies used during the implementation of *Ethos* were fully aligned to identified barriers, seven were partially aligned, and two were not aligned. One recommended strategy which was used during the implementation of *Ethos* (use advisory boards and workgroups) had no specific Level 1 or Level 2 matched barriers and so could not be assessed. The assessment process is depicted in full in Supplementary File 4, with an abbreviated version shown in Table 3 displaying alignment of each strategy to one of the matched barriers for illustrative purposes.

**Table 3:** ERIC strategies used during the implementation of the program and their degree of alignment with barriers

ERIC Strategy	Operationalisation by program implementers	Illustrative example		
		CFIR construct matched to strategy	Description of barrier in context	Alignment between strategy and barrier in context
Conduct educational meetings	Educational meetings and training were held with the different stakeholders (line managers, executive, peer messengers, and all other staff).	Goals and Feedback	Broad goals but lack of clarity about what the program would achieve, perpetuated by the dearth of feedback at both an individual and organisational level on usage of *Ethos*.	Partially aligned - meetings were held, but not on an ongoing basis and not using content from the messaging system.
Identify and prepare champions	*Ethos* peer messengers were program champions with evidence of selection by senior staff and training and support provided to them.	Learning Climate^a^	Hospitals were viewed as frequently hostile and punitive when issues were raised. Spurious focus on quality improvement.	Aligned – Peer messengers wanted to contribute to quality, safety, and workplace culture, and were professional in delivery of feedback that encouraged self-reflection, not blame.
Develop educational materials	An *Ethos* procedure document, as well as promotional materials, were developed for staff and went through several versions.	Access to Knowledge & Information^a^	Training not accessible to all, visibility of *Ethos* declined over time and specific information on the reporting process was inadequate.	Aligned – a range of materials were developed from detailed program descriptions to promotional screensavers and went through multiple iterations as required.
Create a learning collaborative	An inter-hospital *Ethos* working group was established to share challenges and issues of those implementing the program.	Networks & Communication	Poor communication vertically and horizontally, despite procedures for consultation. Networks characterised by lack of transparency, hierarchy, and tribalism.	Not aligned – Issues occurred within each hospital, while collaborative work occurred at the Group level.
Develop a formal implementation blueprint	The program information and implementation plan laid out the aims/purposes of *Ethos*, scope (all staff), and included a high-level timeline for implementation in each hospital.	Goals & Feedback	Broad goals but lack of clarity about what the program would achieve, perpetuated by the dearth of feedback at both an individual and organisational level on usage of *Ethos*.	Partially aligned – Blueprint included only broad goals and strategies. Performance measures and plans for refining the plan were not specified.
Promote network weaving	The program information and implementation plan mentioned the plan to continue “to engage external stakeholders”.	Networks & Communication	Poor communication vertically and horizontally, despite procedures for consultation. Networks characterised by lack of transparency, hierarchy, and tribalism.	Not aligned – poor communication, tribalism, occurred within each hospital, while networking was done externally or at a Group level.
Distribute educational materials	Program materials promoting *Ethos* including posters and screensavers distributed. FAQ and web portal created. Multiple versions created over time.	Access to Knowledge & Information	Training not accessible to all, visibility of *Ethos* declined over time and specific information on the reporting process was inadequate.	Partially aligned – materials distributed initially, but not effectively over time.
Recruit, designate and train for leadership	Both line managers and senior hospital leaders undertook specialised training to support their involvement and leadership of *Ethos*, and some had designated roles.	Leadership Engagement	Staff perceived leaders as not accountable with a poor track record of addressing unprofessional behaviours. Senior leaders were supportive of *Ethos* but not core implementers and not always knowledgeable on the program.	Partially aligned – strategy addressed engagement with the program more than leadership accountability issues.
Assess for readiness and identify barriers and facilitators	Assessment of readiness for implementation was planned for each site 4–6 months prior to implementation.	Knowledge & Beliefs about the Intervention	On balance beliefs were distrusting, sceptical or measured in their view of the program and how effective it could be.	Partially aligned – limited detail on how assessment conducted, and lack of evidence to suggest it adequately evaluated knowledge and beliefs of staff prior to implementation.
Identify early adopters	The independent review examined the process of adoption at two sites first to implement *Ethos* and learned from their experience.	Knowledge & Beliefs about the Intervention	On balance beliefs were distrusting, sceptical or measured in their view of the program and how effective it could be.	Aligned – from analysis of early implementing sites, identified a range of misconceptions about the program and how it worked, recommended improvements.
Use advisory boards and workgroups	*Ethos* Action Plan Working Group created with both internal *Ethos* program leads from each hospital and some external academics. In a series of formal meetings, oversaw refinements to the program following Internal Review.	*No individual barrier with Level 1 or 2 endorsement for this strategy*	N/A	N/A
Involve executive boards	The program had the full support of the Group executive, and at a hospital level, a member of the executive was designated the *Ethos* sponsor. Engagement sessions were run with hospital executive and senior leaders so they could support the program.	Leadership Engagement	Staff perceived leaders as not accountable with a poor track record of addressing unprofessional behaviours. Senior leaders were supportive of *Ethos* but not core implementers and not always knowledgeable on the program.	Partially aligned – addressed program engagement but not accountability issues with leadership.
Obtain formal commitments	The program information and implementation plan specified the full commitment of the Group’s board and outlined various roles and responsibilities of key leaders and *Ethos* sponsors. Commitments were also made related to the evaluation of the *Ethos* program.	Leadership Engagement	Staff perceived leaders as not accountable with a poor track record of addressing unprofessional behaviours. Senior leaders were supportive of *Ethos* but not core implementers and not always knowledgeable on the program.	Partially aligned – numerous key partners were not in a leadership relationship. Commitment from leadership explicit at a Group but not hospital level.
Purposely re-examine the implementation	The independent review and the researcher-led evaluation of the program implementation were conducted. A working group was established, and an action plan put into place following this to ensure recommendation revisions were adopted.	Design Quality & Packaging	Poor perceptions of the messaging tool and process, scepticism about anonymity, dislike of the lack of “natural justice”, confusion about the inclusion of both positive and negative feedback in the one system.	Aligned – evidence of materials and strategies being revised in light of new information and recommendations.

^a^Endorsed by experts ≥ 50% (Level 1) of the time as an implementation strategy that addresses CFIR determinant. Otherwise endorsed at 20-49.9% (Level 2) [25]

*N/A *Not applicable

## Discussion

Our study adds to emerging literature on the implementation of programs to address unprofessional behaviour and improve hospital culture. We found barriers in the quality of the design of the program, enablers in a tension for changing the acceptance of unprofessional behaviour in healthcare, and mixed determinants in the complex and conflicting impressions of the culture within implementing hospitals. Program implementers had used a range of appropriate implementation strategies, although in some instances they were not operationalised to fully address the contextual barrier.

The inner setting was the most influential CFIR domain in the implementation of *Ethos*, perhaps because the program, which focused on organisational culture and communication between staff, targeted some of these constructs directly for improvement. Similar issues were found by McKenzie, Shaw [21] in their study of the implementation of a professional accountability program; they identified that recognition of cultural problems and unprofessional behaviour enhanced implementation, while a lack of accountability among leadership for their own behaviour limited program delivery. In both studies, the prioritisation of confidentiality in the design and use of the messaging tool had some negative impacts on program implementation. Here, it created a closed system in which subjects of *Ethos* messages felt unable to defend themselves against ‘accusations’, while individuals using the system to provide feedback about a co-worker’s behaviour were not followed up about if and what action had been taken.

Unlike Pichert, Moore [16], we found no difficulties in recruiting peer messengers, many of whom volunteered for their role. This may have been due to *Ethos* being open to all staff, not just doctors, which expanded the pool of potential messengers, though representation across professional groups was still required. Some of our enabling factors point to the importance of the context in which the program was implemented, particularly its ‘Structural Characteristics’ as a charitable values-based organisation, which reinforced *Ethos* as a culture change program about enhancing respect among staff. Other enabling features such as ‘Engaging – Champions’ and ‘Reflecting and Evaluating’ were evidenced as specific implementation strategies in the second part of our analysis: ‘Identify and prepare champions’ and ‘Purposely re-examine the implementation’, respectively.

Although there are few studies on professional accountability programs, Jones, Blake [37] synthesised factors affecting the implementation of speaking up interventions in thirty-four studies in healthcare. They identified a core theme of workplace culture, including hierarchy and interdisciplinary factors, which maps to numerous inner setting determinants in our study, including ‘Networks & Communications’, ‘Structural Characteristics’, ‘Leadership Engagement’ and ‘Culture’. Together these findings suggest the importance of considering this domain when implementing similar programs.

Our analysis of strategies used during the implementation of *Ethos* provides a novel contribution to research on these programs, and implementation research more generally [29]. Despite not explicitly using theory, implementers of *Ethos* employed a range of general implementation strategies that have been endorsed by experts as suitable for addressing barriers present in their setting. This suggests implementers had a good working knowledge of implementation practice and some appreciation of barriers likely to be encountered in implementing this program. However, the ways in which implementers tailored these strategies did not always adequately address the nuances of contextual barriers. Alignment was poorest for addressing ‘Leadership Engagement’ and ‘Networks and Communication’ challenges, and strongest for targeting ‘Design Quality and Packaging’ and ‘Access to Knowledge and Information’. The latter two relate to the intervention and how it was delivered, while the former are more global characteristics of the organisations implementing *Ethos*. This suggests frontline implementers may have greater capacity to address barriers that are immediate and proximal to program implementation, rather than distal, longer term, but no less, influential determinants. Hence, implementation theory may be particularly useful for identifying, understanding, and addressing complex barriers [30].

### Implications

Our research, together with previous research on peer accountability and speaking up programs, [21, 37] highlights the importance of the inner setting in implementation. Accordingly, expending time and effort to improve poor or superficial communication (‘Networks and Communication’), or unaccountable leadership (‘Leadership Commitment’), prior to implementing a program such as this is important to foster hospital staff understanding of, and confidence in, the intervention. Targeting the latter might also act as a means of leveraging the widespread recognition of the problem of unprofessional behaviour (‘Tension for Change’) which was tempered to some degree in our study by a scepticism that leaders would not, based on previous experience, follow through on addressing them. Arguably a whole-of-hospital program such as *Ethos* is a lever for fostering greater commitment and accountability among leaders, so long as mechanisms for ensuring this are communicated.

The determinants we have described (Table 2, and Supplementary File 3) provide a starting point and may assist in expediting the process for identifying barriers and enablers in future efforts to implement similar programs. For example, one could prioritise the assessment of influential aspects of the inner setting, (e.g, [38]) as well as other determinants found here, making use of limited resources where maximum benefit can be gained. Our extraction of implementation strategies highlights how these have been operationalised to address barriers, which might be useful in the future design of implementation plans for professional accountability programs. Overall, results signify the importance of using theory prospectively to gain a detailed understanding of factors that will impact on the implementation of a program, utilising these insights to select candidate implementation strategies and then operationalising these strategies in a way that adequately addresses the barrier. An example of how this could be undertaken is provided in Supplementary File 5. In practice, an assessment of readiness for implementation is likely to elicit a range of barriers, enablers, and mixed determinants, just as our study has.

### Strengths and limitations of the research

Triangulation of multiple data sources from many influential stakeholders over an extended period was a strength of this study. However, use of naturalistic data and datasets not originally collected to explore implementation determinants may have affected results. It may have skewed findings to a greater preponderance of barriers because data collection tools were focused on negative aspects of the workplace (e.g., the baseline survey assessed unprofessional behaviour). Furthermore, because the CFIR did not guide data collection, a lack of data for some constructs does not definitively mean they had no impact on implementation, only that this was not salient in these data. Standardisation of data analysis using an implementation determinant framework, however, means we can target these specific constructs in future research, as well as further examining the ones found to be more salient here. In turn, standardisation facilitates comparison of these findings with other research on culture change, speaking up and professional accountability programs.

Finally, the COVID-19 pandemic was a spectre influencing the late stage of the implementation of this program and may in part explain the diminishing ‘Relative Priority’ and the view that ‘Access to Knowledge and Information’ decreased over time. Despite this not being explicit in the data, anecdotal reports from the Group indicate the hospitals sought to prioritise the core business of patient care during this time.

## Conclusion

This is the first multi-data source, theory-based examination of the implementation of a professional accountability and culture change program. Findings demonstrate the importance of the inner setting in influencing implementation, particularly the use of goals and feedback, and having a learning climate and tension for change. Moreover, the analysis demonstrates the value in using implementation theory to not only facilitate an understanding of how a program has been rolled out, but to more effectively address barriers to implementation in complex settings like hospitals. We have established a framework for use in future professional accountability program implementations to facilitate comparisons. Culture change is difficult, and it is fundamental to maximise the use of limited resources with the deployment of implementation strategies tailored to the specific context. Our research begins this process.

## Supplementary Information

**Additional file 1.**

**Additional file 2.**

**Additional file 3.**

**Additional file 4.**

**Additional file 5.**

## Data Availability

Some of the datasets generated and analysed during the current study are not publicly available due to ethical restrictions upon the reuse of data. Other datasets are available from the corresponding author on reasonable request and with appropriate approval.
