# Supplementary material for: Retrospective analysis of factors influencing the implementation of a program to address unprofessional behaviour and improve culture in Australian hospitals
Source: BMC Health Serv Res. 2023 Jun 7;23:584. doi: 10.1186/s12913-023-09614-1 (PMC10244846; doi:10.1186/s12913-023-09614-1)
Supplement: Supplementary file 3 — Additional file 3. [file 12913_2023_9614_MOESM3_ESM.docx]

| Domain | Construct | Summary in study | Presence in data | Exemplary quotes | Determinant type |
| --- | --- | --- | --- | --- | --- |
| Intervention Characteristics | Intervention Source | Key stakeholders and innovation participants acknowledge Ethos has having an external source. | minimal | "The Ethos accountability pathway has been developed based on based on the principles of the Vanderbilt Promoting Professional Accountability model (Hickson GB et al. A complementary approach to promoting professionalism: Identifying, measuring and addressing unprofessional behaviours. Acad Med. 2007; 82:1040-1048) and other similar models." 1. Program information and implementation plan  "…imported American system" 3. Baseline survey of all staff (Hospital 1) |  |
|  | Evidence Strength and Quality | Some mentions that the model was validated and the innovation had an evidence base, but weakly represented | minimal | "A validated, tiered accountability pathway … " 1. Program information and implementation plan  "Yeah, there are some things in it that are evidence based. And I probably need to see them working a bit more and probably understand them a bit more…" 4. Interviews with senior hospital leadership (participant 140) |  |
|  | Relative Advantage | Some stakeholders and participants indicated that Ethos was not the best solution to the issue of poor culture or unprofessional behaviours highlighting that quality leadership was paramount, while a couple of senior leaders suggested it was better than other programs particularly as it focused on early intervention. One indicated the anonymous nature made it suitable for when there was a power imbalance between two people. | moderate | " I don't think Ethos is the answer to this problem - I appreciate and support improving culture - but we are really just working around the fact that no one in this organisation exhibits the authority or will to want to tackle this. This is the job of a manager and not an Ethos team." 3. Baseline survey of all staff (Hospital 1)  "...overall a reasonably good tool, although I’ve thought this for a while and we just spent three days on our leadership courses, and the investment in their leadership and that capability is probably a much higher priority, if you were to really shift the culture around being able to speak up and not fearing, so that patients outcomes were safer." 4. Interviews with senior hospital leadership (participant 140)  "It's about that safety and confidence aspect. I think it does a better than most other things I’ve seen, if it’s possible..." 4. Interviews with senior hospital leadership (participant 150) |  |
|  | Adaptability | n/a | n/a |  |  |
|  | Trialability | n/a | n/a |  |  |
|  | Complexity | Brief mentions that the process for using Ethos Submission System is straightforward, or challenging from a participant without capacity to use a computer. | minimal | "I think what's been really good as we put it as an icon on our desktop, and it's pretty self explanatory, like it takes 30 seconds to report. So if you can click a mouse, then you can do it." 4. Interviews with senior hospital leadership (participant 210) |  |
|  | Design Quality and Packaging | Stakeholders and innovation participants highlighted weaknesses in the way the innovation worked and was packaged. Numerous people suggested the supposed anonymity of Ethos messages was impossible because a message required detail. Others said the anonymity was one sided. The anonymity also precluded a closing of the loop with people making Ethos submissions not getting details about how the message was received by the other party and hence unable to gauge its effect. The presentation of the innovation was also criticised as being more formal than originally intended and unbalanced, weighted toward the negative rather than positive Ethos reporting. Indeed, some indicated that the inclusion of both positive and negative submissions within the same reporting system was inappropriate, questioning why positive feedback could not just be provided publicly or in situ. | high | "The terminology used within the Ethos program implies a formality which is not intended and could be improved." 2. Independent review of Ethos program in two hospitals  "The Ethos idea is flawed. I'd like to make some comments about specific staff, but in order to do so you need specific examples for it to actually mean something. This completely unblinds the anonymity of the process! I am afraid of being victimised for speaking up about the behavior of staff, especially those more senior than I. On the flip side, someone who was "Ethos-ed" made a valid point: it's effectively an accusation based on feelings, and there is no capacity for the accused to defend themselves or give a right of reply. " 3. Baseline survey of all staff (Hospital 1)  "... (I'm) a bit curious about the positive reporting, which I think is a fine thing, and it's probably done, maybe to sort of… Actually I’m not too sure why it’s been done now that it's been in for a little while because we're probably expected to see, and understand the confidentiality about the negative reports and how that gets dealt with. But it seems like it's a waste of positive feedback for it to kind of go into a system and maybe a message goes back but then I don't know who actually finds out about it." 4. Interviews with senior hospital leadership (participant 110)  "One of the biggest challenges I found… is that someone puts in an Ethos, and then they get the email to say it’s dealt with. And then they go, what does that actually mean? I haven't got closure… 4. Interviews with senior hospital leadership (participant 130)  "… there's no right of reply... I’m still not clear on the impacts of that. I have seen some messages delivered that are clearly vexatious and, and we've heard about those... " 4. Interviews with senior hospital leadership (participant 140)  "…there're the great examples in theory of where that is, anonymous is totally appropriate... But we know with accountability it allows people to not have to back up what they're saying or in fact take personal responsibility for some of the low-level workplace conflicts to resolve." 4. Interviews with senior hospital leadership (participant 170)  "... and the way it is on here, the help sheet, is when and how to report. So, the last point is you can report positive and negative behaviour, but the first three parts are more about if you witness negative behaviour, ill-equipped equipment, speak up, report it, it's safe and confidential." 6. Interviews with middle managers | barrier |
|  | Cost | When cost was mentioned it was in relation to Ethos being a waste of money. These were coded to Relative Advantage or Relative Priority as appropriate. | minimal |  |  |
| Outer Setting | Needs and Resources of Those Served by the Organisation | In relation to this program, which targets staff not patients, it was the needs and resources of hospital staff that were considered. Survey responses from staff across hospitals indicated that their senior leaders were disconnected from their work and work issues. There was reports of a lack of consultation by hospital executives, and unwillingness to prioritise their need over other issues, e.g., revenue. However, many of these comments were coded to Leadership Engagement. | minimal | "Executives don't consult and/or ignore the people actually doing the job before making changes. This usually leads to unreasonable and unrealistic expectations, inefficient work, lack of storage and more complications." 3. Baseline survey of all staff (Hospital 6)  "It seems that there is no understanding from executive the pressures that staff are put under when unplanned leave, planned leave and vacancies are not covered and filled and recruitment is delayed. Staff are often doing loads of unpaid overtime as they feel that very vulnerable patients will be disadvantaged if they don't. Being told by management to 'just do what you can' and 'make sure you go home on time' is lip service." 3. Baseline survey of all staff (Hospital 1) |  |
|  | Cosmopolitanism | Brief mention that Ethos will involve sector wide relationship building. | minimal | "The Ethos program also includes the development of relationships across the sector, recognising that culture change of this magnitude cannot be undertaken in isolation." 1. Program information and implementation plan |  |
|  | Peer Pressure | One source acknowledged that similar professional accountability programs were being adopted within other hospitals in Australia and internationally. | minimal | "Programs adopting Vanderbilt and Ethos principles are being implemented at present in an expanding number of health services across Australia… Other health services are using this and similar programs and they are considered successful." 2. Independent review of Ethos program in two hospitals |  |
|  | External Policies and Incentives | A couple of sources alluded to scrutiny of bullying, harassment and unprofessional behaviour across the healthcare sector and noted a similar system wide program. Moreover, some stakeholders noted a broader cultural shift in the acceptability of these sorts of behaviour. However, one stakeholder suggested the public organisation mentality made their organisation more willing to tolerate unprofessional behaviour, rather than taking a hard-line stance. | minimal | "External scrutiny of the health sector has indicated that the sector is failing to respond effectively to entrenched cultures and behaviours that put the health and safety of staff and patients at risk." 1. Program information and implementation plan  "It's gonna be very hard to distinguish between the impact of Ethos and the change of community expectations about behaviour... The atmosphere is changing so quickly. Colleges of Surgeons, all these people. Everyone is, no one is tolerating bad behaviour. You know, Me Too." 4. Interviews with senior hospital leadership (participant 220) |  |
| Inner Setting | Structural Characteristics | There was some variability across stakeholders perspectives dependent largely on the hospital site in which they worked, including whether it was public or private, large or small, and noted things like increased focus on cost cutting or money making, bureaucracy, and how inclusive the hospital was of new staff. However, overarchingly participants drew attention to the value-based nature of the wider organisation, with some also highlighting that it had a developed from charitable and Catholic sensibilities. Some suggested that this contributed to a positive working environment and made the organisation a desirable place to work, both attracting and retaining staff. However, some innovation participants indicated that the widely promoted values of the organisation were not always reflected in conduct within the hospital or the behaviour of leadership. | high | "SVHM has a long legacy of living our values through professional practice" 1. Program information and implementation plan  "Staff need to be aware that they are in a Catholic private hospital and there is an expectation of commitment to the values and expectations of moral behaviours." 3. Baseline survey of all staff (Hospital 6)  "Much of the behaviour examined in this questionnaire (especially in regards to the hostile interactions between staff) are in my opinion, the result of a lack of resources being forth coming from the private business models the hospital now employs." 3. Baseline survey of all staff (Hospital 5)   "I like the emphasis on values in my workplace left by the Nuns who began the organisation." 3. Baseline survey of all staff (Hospital 5)  "...the majority have utmost respect for their colleagues and we choose to follow the mission, vision and values - it is the major reason I work at my place of work." 3. Baseline survey of all staff (Hospital 2)  "[The hospital] through its good name recruits high-quality staff, particularly new graduates and younger staff. So there exists a great sense of camaraderie and goodwill among hospital staff in general." 3. Baseline survey of all staff (Hospital 2)  "I think it is an attractor, I think it's part of our whole staff engagement strategy that people, you know work here, a little bit because of the mission. But obviously they work here because you know they work in public health work, or want to work in public health, and have a particular craft group or specialty type interest. And so that's why I think people work here. People tend to stay here a long time. So I think [it’s] is a bit of an attractor and it's also a retainer." 4. Interviews with senior hospital leadership (participant 130)  "... it's a values based organization and like other religious hospitals I've worked in, (we) ... wear our values more on our sleeve than secular organizations. So that's nice... the mission of the charitable mission is clear here, and the compassionate mission towards the poor and vulnerable is good. The working relationships within the hospital. It's a slightly smaller hospital than I'm used to and so that doesn't have quite the same complexity of doctor-to-doctor relationships as I'm used to in a bigger hospital. The overall, the, the nursing staff are quite senior and experienced and that's good quality nursing. " 4. Interviews with senior hospital leadership (participant 220) | enabler |
|  | Networks and Communications | Numerous participants across sites reported poor vertical communication with leadership. There were also mentions of poor communication between professional groups and departments, and between clinical staff and managers. While some suggested that processes for proper communication and consultation were in place, they were not always adhered to. Some participants indicated these communication issue were just to be expected in a large hospital, while others suggested there was a deliberate lack of transparency, hierarchy and tribalism. | high | "There needs to be more open transparent communication between Executive and the rest of the hospital. The secrecy leads to staff unrest, job uncertainty and negativity amongst team members, which therefore impacts staff behaviour." 3. Baseline survey of all staff (Hospital 6)  "Lack of clear structured communication." 3. Baseline survey of all staff (Hospital 5)  "There remains a hierarchy / tribe mentality with doctors / nurses / allied health / other groups treated differently by the organisation depending on their status. This reinforces behaviour with staff." 3. Baseline survey of all staff (Hospital 1)  "Executive staff have been known to change vital policies and procedures without consultation with the staff that will be affected most by these changes." 3. Baseline survey of all staff (Hospital 2)  "There is a consistent culture of withholding information from clinical staff (despite monthly updates which are generally informationless) and of asking clinical staff for input which is then ignored. The opacity with which the hospital is run makes angry, anxiety-driven behaviour of clinical staff more likely." 3. Baseline survey of all staff (Hospital 3)  "All staff members are open for exchanging of information when needed as well as very receptive to any suggestion and issues that may arise during the shift." 3. Baseline survey of all staff (Hospital 4)  "Communication is poor and disjointed between the Management and staff. making change the more difficult" 3. Baseline survey of all staff (Hospital 4) | barrier |
|  | Culture | Highly mixed views on the culture across the group and within each hospital. Stakeholders from some hospitals indicated there was a culture of division, bullying, blame and fear, others suggested their site was a positive, supportive and respectful place to work, though there were also frequent mentions of favourtism in one hospitals. Respondents sometimes mentioned a business or financial focus as driving their organisation, while others highlighted the benevolent values that had historically shaped the organisation, indicating they were still important in guiding behaviour. | high | "Staff are very quick to blame others to avoid taking ownership of mistakes that they make. They are also very quick to blame things that do not go right for patients on peripheral staff." 3. Baseline survey of all staff (Hospital 6)  "I generally have found the environment in which I work to be inclusive, respectful, and extremely pleasant." 3. Baseline survey of all staff (Hospital 5)  "I feel there is a culture of favouritism at the Hospital 5, which is very difficult to cope with. Certain individuals are allowed to behave in any manner they please, but there seems to be insufficient ramifications for them. As somebody who is always respectful and professional, it is very disheartening to see such behaviours be inappropriately dealt with, and means that staff who behave as per the Code of Conduct are given less air time, when they are the ones who deserve air time and praise." 3. Baseline survey of all staff (Hospital 5)  "There is loads of advertising about our hospital values etc. They have absolutely zero of them towards staff if it saves them even a single dollar. Cut any corner and screw over staff as much as possible to save money. That's all they actually care about." 3. Baseline survey of all staff (Hospital 1)  "The overall behavior of staff at [the hospital] is very good - I have worked in the public hospital system for almost 40 years and [the hospital is by far the best institution I have been part of. The values are embodied not just a plaque on the wall" 3. Baseline survey of all staff (Hospital 1)  "It is a closed culture that is focussed on watching your back." 3. Baseline survey of all staff (Hospital 3)  "On the whole, I think everyone is respectful, polite and friendly. Everywhere I go around the hospital and the unit I work in people say hello and have a smile on their face. It is a very friendly environment." 3. Baseline survey of all staff (Hospital 3)  "Because the culture here is, like I said, genuinely, respectful and professional. It is, it should be anchored to and it is underpinned by the mission and values of the organization." 4. Interviews with senior hospital leadership (participant 180) | mixed |
|  | Implementation Climate | Staff participating in the baseline LION survey across hospital sites cited destabilising issues related to frequent changes in management, restructuring and redundancies. Many also highlighted extremely high workloads and bureaucratic processes, with some noting that this impacted on capacity to engage in change. Some mentioned a history of developing programs that then not appropriately implemented or sustained. | moderate | "We are constantly being paraded with new levels of management with no accountability and then there is too much red tape to in fact implement change." 3. Baseline survey of all staff (Hospital 1)  "Often exciting & beneficial programs are developed but implementation is not followed thru" 3. Baseline survey of all staff (Hospital 2)  "Exceptionally inappropriate restructuring processes within a department. Four restructures in a department over a period of less than five years is unacceptable." 3. Baseline survey of all staff (Hospital 4) |  |
|  | 1. Tension for change | Nearly all sources and stakeholders recognised the problem of unprofessional behaviour, which the implemented program aimed to address. However, some stakeholders, particularly among LION survey respondents, raised the view that within their workplace, those in senior positions had frequently tolerated such behaviours in the past and so were sceptical about the extent to which Ethos would or could make a difference in the future. That is, while individually, stakeholders implied that the situation was intolerable, they expected that within their hospital it would continue to be tolerated, particularly by management. During interviews, some senior managers did also minimise the impact of some unprofessional behaviours (as measured by the LION survey), noting that they were low level or subjective. Senior managers also conceded that there had been tolerance of poor behaviour for a long time, but some indicated that this was changing and the implementation of Ethos was a reflection of that. | high | "There is a need to change entrenched negative cultures. The Ethos program provides an opportunity to achieve this." 2. Independent review of Ethos program in two hospitals  "I wonder how badly a staff member would have to behave towards others to actually lose their employment. I have seen some particular staff members behave in a bullying manner for many years with no apparent ramifications." 3. Baseline survey of all staff (Hospital 6)  "Much of the behaviours are very entrenched. Best described as verbal bullying, aggressive and intimidating. Interesting management state they are aware of it and it has been an issue for a long time yet there seems to be very little evidence of tackling the problem." 3. Baseline survey of all staff (Hospital 2)  "I find it appalling that members of the executive as high as the CEO are aware of constant and extreme examples of bullying in this organisation and do nothing about it. These bullies/individuals are still in their roles, still bullying, devastating people's lives and negatively impacting the care we deliver." 3. Baseline survey of all staff (Hospital 3)  "...we know where there’s poor behaviours, that equals poor outcomes for our patients, notwithstanding the poor outcomes for our staff. People tend to buy into that poor outcome for our patients and understand that, so I think the more that we've been able to get that message through, the more people understand that the driver behind the Ethos program, and why ultimately it's important as a tool to, to help us keep an eye on, on behaviours and to help people understand, again, how their behaviours impact, others." 4. Interviews with senior hospital leadership (participant 160) | enabler |
|  | 2. Compatibility | Some sources and respondents suggested that the principles of Ethos relating to feedback, respect and giving people a voice, as well as its processes, where complementary to existing systems and organisational, management and professional development processes. On the other hand, some participants suggested that the giving and receiving of peer feedback was not intuitive. Several respondents were uncertain about the integration of Ethos with existing HR processes; a senior leader further suggested that feedback provided through Ethos might be better fielded by HR processes in some instances and another noted that because of the anonymity of Ethos, data insights provided by it were not well integrated with other information. One interview participant mentioned that sometimes Ethos and an additional organisational system might be used to report the same issue leading to parallel investigations. Hence, there was the potential for confusion among staff about which system to use and whether something had been entirely resolved or not. | high | "The foundations of giving and receiving feedback are core to employee development." 2. Independent review of Ethos program in two hospitals  "I think Ethos is wonderful and I support it wholeheartedly however it is the process after Ethos that worries me as we do not seem to have the appropriate framework in place to support a culture change program such as this. Not from a HR perspective at least." 3. Baseline survey of all staff (Hospital 1)  "But professional feedback in the medical world is very hierarchical, so it’s, that feedback is not peer either, but it's very hierarchical and it's, and it’s, you can see it in the younger doctors, it’s, though they might be not used to it, it's done in fear so when they receive it... even though they kind of receive that feedback, they take it from a position of, of failed." 4. Interviews with senior hospital leadership (participant 140)  "I'm getting data points from other things like, be it, poor quality indicators, poor financial indicators, poor other HR practices, annual leave, management, sick leave or whatever it might be. And it would be useful to marry that together with Ethos and be able to put the whole picture together and say well okay, we've got a problem here and let's dig a little deeper into understanding that, but because I don't have any visibility and nor my general managers have that visibility it just makes it more, it makes it more difficult." 4. Interviews with senior hospital leadership (participant 160)  "I don’t think Ethos is set up to do that so I guess to answer your question, the connectivity between the manager, the person they deliver the message. And really, HR support of well, what does this mean, what does this look like. Probably for me yeah, it's very person dependent, i.e., me, drawing the dots together and saying to people... I will take off an Ethos hat and put on an HR hat and have a conversation with that manager because I am concerned that she has some underlying trust issues in her team that we need to help her address because she's not going to get where she needs to go if she can’t address them so I will use it as, that’s not Ethos, that’s HR 101... bullying and harassing behaviour I think has to be dealt with through a HR process... I think you have to go in a formal HR process…I’m desperately trying to build a zero-tolerance culture of bullying and harassment, Ethos is too closed a circuit to be able to do that at the moment." 4. Interviews with senior hospital leadership (participant 170)  "The system seemed to be a formalisation of the existing feedback process already in use in the department, and I welcome initiatives that give structure to an organic system that is already working." 5. Survey of Ethos peer messengers | mixed |
|  | 3. Relative Priority | There were mixed impressions about the relative priority of Ethos, with some participants from the LION survey indicating there were other or bigger issues that needed to be attended to by their hospital, before or instead of Ethos. However, many Ethos messengers were very positive about the program, indicating they believed in its principles and became involved formally to see that it was a success. Middle management reported that there was not a sustained interest in the program, that it had not received the level of promotion and concomitant with that, staff were busy and occupied with other matters, and did not see any required training in the program as a priority. | high | "Whilst I think this is a worthy program, it appears mostly designed to identify and correct unpleasant behaviours. Whilst I have not experienced many specific 'unpleasant behaviours', I do think that overall improved communication within the hospital would make an enormous difference in terms of efficiency and patient outcomes (eg. medical specialists co-managing patients, goals of care of medical and nursing staff, priorities held by hospital administration/finance as opposed to those seen as important by medical staff etc...)." 3. Baseline survey of all staff (Hospital 1)  "I haven't used Ethos because I don't see the issues as being individual behaviour, but a problem with the culture of the organisation. There is a culture of blame and responsibility shifting and not customer service (towards the staff). HR and other corporate services including finance, payroll and IT don't treat staff like customers but like problems." 3. Baseline survey of all staff (Hospital 1)  "Does 1% of staff, perhaps not always behaving well equate to a 'new system' introudction, or will this new system (ethos) encourage an over-critical (looking for things that are not really there) mind set." 3. Baseline survey of all staff (Hospital 1)  "I have thought it is a beneficial program to help staff who feel they have no other course of action to be heard and their complaints to have a pathway to action." 5. Survey of Ethos peer messengers  "All the staff just flick through it. Do they actually read it? Do they engage in it? Not sure. Then you hear very little about it as well. I’m not sure whether – I feel like it’s a program that it’s spoken about, but not really spoken about." 6. Interviews with middle managers  "Ethos is yet another thing that’s been rolled out. The Group famous for introducing new programs bang bang bang and nothing really happens." 6. Interviews with middle managers | mixed |
|  | 4. Organizational Incentives and Rewards | Few sources touched on incentives, although it was mentioned a couple of times that Ethos peer messengers, champions of the program, did not receive tangible rewards for performing their role. At the same time, some mentioned professional development opportunities in taking part. | minimal | "Sometimes I feel like we're just getting people to do a dirty work. It’s not an easy job. And, that you know, I’ve already seen some backfires on messengers and not very nice for them. And they're not being paid for what they do, whereas I’m being paid to take the heat." 4. Interviews with senior hospital leadership (participant 220) |  |
|  | 5. Goals and Feedback | Broadly, stakeholders indicated that the goals of the organisation, in code of conduct, policies and values, were clearly communicated, but that supporting them through monitoring, feedback and performance management was limited and skewed toward negative behaviour. Some senior leaders did report feeding back results of the baseline survey to their heads of department and doctors on unprofessional behaviour which was used to both evaluate and support Ethos. Specifically for the Ethos program, goals about the type of culture desired were articulated at the outset and the program; however, some stakeholders reported that what the program aimed to achieve was inconsistently communicated with regard to positive feedback and the inclusion of all staff. Furthermore, numerous sources highlighted that the nature of the reporting system precluded those who made an Ethos submission getting information about how the feedback had been received by the person they reported. The confidential nature of the system also made it difficult to report on the outcomes of using Ethos on an organisational level, even for positive reports that were supposed to celebrate exceptional behaviour among staff. This information was not communicated hospital-wide nor to leadership in any consistent way; though some hospitals did have strategies to report on utilisation of Ethos it was unclear whether these were effective. There were some indications that the goals of a system focused on addressing and feeding back information about negative behaviours might be incompatible with acknowledging and celebrating positive behaviours. | high | "[There is] A need to improve communication around positive Ethos reports and exceptional role models...Positive feedback and positive reports are not widely communicated or promoted, creating a perception that most Ethos reports are negative (however, in the previous 12 months at Hospital 1, 40% of all Ethos reports were about positive behaviour). " 2. Independent review of Ethos program in two hospitals  "Yes, I have already put in an ETHOS negative report about treatment to me from a staff member but have received no acknowledgement or feedback to date." 3. Baseline survey of all staff (Hospital 1)  "I've been through this many times where people will say, well what happened? I go, I can’t tell you, cos we’ve dealt with it, we’ve dealt with it appropriately, and you have to trust that we’ve managed it, and you know obviously if it happened again we need to know, but yeah, I've had that with Ethos... it's really challenging... you have to trust us that we've dealt with it. There's no detail. So that's really challenging. I think it's, I think the philosophy of Ethos is good. As for how effective it is, in terms of this, err, I’d question how effective it is probably." 4. Interviews with senior hospital leadership (participant 130)  "...they gotta work out a way to report back to the business of what we've done about things. So what is the point of it. And that's what we get lost with, have we changed behaviours, have we managed difficult people out?... There’s no report out to the business. Even the executive don’t get reports. Going to start changing that, but no, no that’s a problem." 4. Interviews with senior hospital leadership (participant 170)  "I think the program needs to evolve... how are we feeding back to staff about where we're at, to where it's at. It's like a news survey, you know, you do a survey to collect information, people often want, what’s the results of the survey? They need to, we need to close the feedback loop. It’s basic behavioural change, you know. You don't change what you don’t measure. You need the feedback to complete the work... I think it's great that it's not just seen as a negative program." 4. Interviews with senior hospital leadership (participant 180)  "I don’t know what they want to achieve out of the ETHOS programme. It … was sold to us like that, it was like, well, we're not nursing staff, we're not doctors, what does this system have to do with us? So, we didn’t see the point in using it, if that makes sense... when we discussed it, it was looked as like a dobbing tool... So, I think there was some confusion about what is this actually programmed for." 6. Interviews with middle managers | barrier |
|  | 6. Learning Climate | Numerous respondents to the baseline survey across hospitals implied that their organisation was hostile, punitive or ineffective in dealing with reports related to e.g., safety and clinical issues. Some suggested that the focus on evidence-based practice and quality improvement were limited and that many staff did not feel able to speak up. However, some leaders mentioned that they were in a process of transitioning their organisation to a greater focus on improvement and were using various forms of data (including Ethos) to monitor quality issues in their workplace. | high | "The picking out of mistakes on the workplace is probably the most annoying thing about working at the Hospital 5. Each week we are sent a weekly bulletin with all the things the nurses are doing wrong then at the bottom of the email the weekly bulletin says - keep up the good work! There is focus on mistakes daily! [Incident reporting tool] is meant for to be about looking at systems and processes and how to improve things so nurses can get back to the bedside to provide care yet we are reminded daily to complete hourly rounding sheets and other punitive paperwork that takes us away from the bedside." 3. Baseline survey of all staff (Hospital 5)   "People shift blame. Roles are blurred. Instructions are unclear and when you try to sort something out you are verbally attacked (or emailed) if something is not done 'correctly' even though you tried to seek out the 'correct' process. No chance of allowing human error after mountainous loads of data entry or work being done under immense time pressure or whilst juggling multiple roles." 3. Baseline survey of all staff (Hospital 1)   "There is a culture of blame and finger pointing if any errors occur in the workplace. This does not foster an open and supportive work environment." 3. Baseline survey of all staff (Hospital 2)    "Executive members generate a culture of bullying and finger pointing, they are reactive rather than pro-active and unwilling to listen and act on clinician's thoughts and knowledge for system improvement. There is no real acknowledged appreciation or gratitude for clinicians work." 3. Baseline survey of all staff (Hospital 3)   "...we’ve taken a journey approach to how we embed improvement thinking. Yeah, so it started two and a half years ago, and the idea of the first two and a bit years, two years, is to build our foundation, so how we look at problems. And that's not just technically how we assess them and break them down, find the root cause and counter measure them. It's about opening people up to, showing us and being transparent around failure, and then not... in the last six months that it's taken off significantly... One of the key things is that, [staff] feel freer to talk about the failure, to speaking up is starting to happen." 4. Interviews with senior hospital leadership (participant 140) | barrier |
|  | Readiness for Implementation | n/a | n/a |  |  |
|  | 1. Leadership Engagement | Limited information about leaderships' commitment specifically to the Ethos program, except to suggest that commitment to the program indicated a desire to improve the hospital culture and address unprofessional behaviour that might have previously gone unresolved. However, there was a great deal of data in baseline survey responses about poor accountability among leadership, lack of transparency and reports that they did not address bullying or unprofessional behaviour in general and were disconnected from frontline workers. Hospital leaders interviewed for this study were broadly supportive of Ethos but varied in their level of knowledge of the reporting system and its use, and how to make best use of the program. | high | "The SVHA Board have fully committed to the implementation of the Ethos program across SVHA." 1. Program information and implementation plan  "Members of the executive are perpetrators of bullying themselves, it seems implausible to me that they could be the architects of cultural change in this regard." 3. Baseline survey of all staff (Hospital 3)  "The executive Management style has gone from a consultative style to one of leadership by fear , disempowering people by making everything secret and keeping staff in the dark. Where there had been trust between management and the staff now there is a commanding authorative style and fear." 3. Baseline survey of all staff (Hospital 4)  "I think there's a lot less tolerance now and so I think there's a much greater willingness for those things to be addressed. I’d suspect though, I'd probably say that that's certainly true of our new current CEO. But yeah, I think the real test would come if it's someone who is quite important for the organisation and whether there'd be hesitation or not or willingness to accept the implications. I think there probably would be. Now, even perhaps 12 months ago perhaps not necessarily or no one knew enough about it or that, but there's a lot less tolerance." 4. Interviews with senior hospital leadership (participant 110)  "So the Ethos tool which helps de-identify feedback and helps a select group deal with that feedback. So that's an opportunity. So I think that, yeah, the key thing for us now is what are our actions. As an exec team and other leaders are [inaudible] with our teams, what are the actions. So I think that, that I've not really been here long enough or spent enough time reading the report to absorb and things like that, but I will. Yeah, so. but I don’t think you can have one specific action I think it's gonna have to be, I think around engagement. And within that, behaviours, around leadership." 4. Interviews with senior hospital leadership (participant 130)  "I don’t think it’s embedded enough in the executive team, and you know I find a big hurdle is what's been successful, the one, the two examples I've done of it is, but it's linking up with HR right.." 4. Interviews with senior hospital leadership (participant 170)  "Importantly, it also indicated to us that management was taking a meaningful interest in the culture of the workplace at every level and seeking to deliver some real changes; I have been waiting for something like this to happen and wanted to give it my best support." 5. Survey of Ethos peer messengers | barrier |
|  | 2. Available Resources | Limited information available in the data, except with regard to the need for additional resources for peer messengers including training for completing their role and administrative support to organise meeting and delivering feedback, and finding private space to carry these conversations out. There were also some comments related to using and accessing the online submission tool for Ethos related to some staff not having access to computers, the fragility of IT systems, as well as potential illiteracy among some staff. | moderate | "[There is] A need to boost skills among Ethos messengers to better support recipients of a negative Ethos message." 2. Independent review of Ethos program in two hospitals  "I mean, our IT capability here is extremely fragile. And our capability is at a very low level, right now. Senior doctors are not able to access hospital intranet or email from outside, they’re having all sorts of troubles, you know. I mean that's, that's a basic thing that needs to be fixed because we just can't, can't seem to get it right I’m afraid." 4. Interviews with senior hospital leadership (participant 190) |  |
|  | 3. Access to Knowledge and Information | The Ethos program manual described how different levels of management, and all staff would be variously trained to use the reporting tool, and understand the accountability pathway, expectations for staff behaviour and delivery of Ethos messages. However, other sources suggest the information was insufficient, not easy for everyone to access and did not provide an ongoing basis to support adoption. Peer messengers reported requiring more knowledge, training and support to handle their roles. Additional information about the program online and in promotion Hospital 5ials was largely suggested to be inadequate, with numerous comments that further promotion or visibility of Ethos was necessary. Even senior hospital leadership at some sites reported not having access to enough information about the utilisation of Ethos, and the independent review of the program noted multiple areas were better clarity and increased transparency were required about what happens through the process of reporting, triaging and delivering an Ethos message. Moreover, some suggested that promotion Hospital 5ial was skewed toward the identifying and reporting negative behaviours. A minority of participants, however, suggested that promotion was adequate and there was a good level of awareness of Ethos. | high | "The target audience for this program of cultural change is all staff at [this hospital]. This will require a flexible approach including a CEO led communications strategy and a combination of face-to-face and online training for line manager and peer messenger groups and a program of awareness raising and skills training for all staff." 1. Program information and implementation plan  "[Required:] More clarity around what happens when a negative Ethos message is received... Improve transparency and education around the triage process using examples of reports that have been progressed and those categorised as un-actionable... Provide continued education about the Ethos program’s objectives and examples of appropriate and inappropriate reporting." 2. Independent review of Ethos program in two hospitals  "Ethos was introduced and i think 1-2 sessions were given explaining this. But if you are part time like I am, then you can't always get to sessions… Pamphlet on the wall at work is really not appropriate as we do not know what it is for. " 3. Baseline survey of all staff (Hospital 1)  "the FAQ or online info in my opinion do not allow staff to be confident to Ethos -this needs education and example profiles to allow people the confidence to report so they can understand the exact process." 3. Baseline survey of all staff (Hospital 1)   "Now I think it's open, it's transparent, it's been well circulated throughout the organization. Everyone knows what Ethos is, which is really good. And that was my test after a month I’d just walk round and go, so tell me about Ethos and if people could articulate it, then it's well embedded." 4. Interviews with senior hospital leadership (participant 210)  "Better education to staff as some still don't believe its effectiveness." 5. Survey of Ethos peer messengers  "I think people have forgotten about the program, and don't readily use it. It needs to be front of mind like [another state-wide reporting program], etc." 5. Survey of Ethos peer messengers  "More visibility for the programme across the hospital... although I acknowledge the impact on COVD on this." 5. Survey of Ethos peer messengers  "there are signs everywhere, in the lifts, and posters about it. " 6. Interviews with middle managers | barrier |
| Characteristics of Individuals | Knowledge and Beliefs about the Intervention | Across the survey open responses, knowledge and beliefs about Ethos spanned highly negative to positive and supportive. On the negative side, numerous comments focused on dislike of how the program works, lack of belief in its effectiveness, distrust in the process, concerns that it may be ill-used or cause more harm than good and worries about confidentiality or reprisal following reporting. The less frequent positive responses touched on belief in Ethos' validity, effectiveness and suitability for addressing the problem of unprofessional behaviour particularly between people with a power differential. Other sources indicated broad support for the goals of the Ethos program but were measured in their view of how effective the program would be, suggesting it was not a silver bullet to address cultural issues and could have unintended consequences. | high | "The overall objectives and intentions of the Ethos program are well supported by all professional groups." 2. Independent review of Ethos program in two hospitals  "Staff regularly threaten each other (jokingly) with "Watch it, I'll Ethos you". Whether this devalues what the program is set out to do." 3. Baseline survey of all staff (Hospital 1)  "[This organisation] overall is a workplace in which respect is paid to staff members by staff members. Programs such as ETHOS will have little impact on altering the rare individuals who act inappropriately in the workplace rather it is a program that allows for allegation's to be made perhaps without merit." 3. Baseline survey of all staff (Hospital 1)  "The ethos program is nothing short of institutionalised dobbing, and will do nothing to address the individuals everyone knows (some of whom are actually part of the ethos promotional Hospital 5ial), who are simply too powerful to force change on." 3. Baseline survey of all staff (Hospital 1)  "I would entrust the Ethos process to action this in a more effective way than our current HR processes have tried to." 3. Baseline survey of all staff (Hospital 1)  "The Ethos programme is counter-productive. It is akin to the Stasi whereby individuals can report others secretly and with no evidence of wrongdoing. It is lazy of the hospital to implement this programme without proper protocols for unwanted behaviour." 3. Baseline survey of all staff (Hospital 1)  "I think Ethos is an excellent initiative to assist small pockets of sub optimal behaviors come to light and understand that it is not acceptable at [this hospital]." 3. Baseline survey of all staff (Hospital 1)   "So I suppose it's one tool, and it's, it's not the whole solution by any means but it's one aspect of an attempt to try and reform the culture of the organization over time and it's while in an industry that's got some probably very well established set cultural norms or previous ones that do need to be challenged and changed." 4. Interviews with senior hospital leadership (participant 110)  "To simultaneously convey the message and to downplay its significance and the relevance of the Ethos program in general. It is a challenge to prevent the Ethos program from causing demoralisation and the development of a toxic, suspicious, resentful culture amongst staff." 5. Survey of Ethos peer messengers  "Staff are afraid to use Ethos as they feel very frightened that the anonymity isn't "real". I have talked with many staff members about this topic and despite my robust response supporting definite anonymity they still do not trust and fear they will lose their job." 5. Survey of Ethos peer messengers | barrier |
|  | Self-efficacy | There was minimal data on this construct in isolation, except to mention that reporting unprofessional behaviour through Ethos took courage and, particularly if there was a supervisory or hierarchical relationship, not all staff would feel secure to do so. These issues frequently dovetailed with concerns about the anonymity of the Ethos reports, and so were coded elsewhere (e.g., Knowledge and Beliefs, Design Quality and Packaging). | minimal | "...it still takes a person to be courageous enough to report it. And I think depending on the situation there will be people who don't feel courageous enough, even though you've got the opportunity to be anonymous, there are always going to be people that are frightened, for whatever reason." 4. Interviews with senior hospital leadership (participant 240) |  |
|  | Individual Stage of Change | Only a few brief mentions in the interviews with executive leadership of how some staff had taken on board the teachings of Ethos, while others were still resistant to utilising the reporting tool. | minimal | "There are still, there was an issue with a doctor being very rude to someone and department, but they didn't want to take it any further and they weren’t going to, and they were, well if you don’t want to do it officially you can do Ethos, no I'm not doing that, I’m not doing that either. But I still dealt with it because I knew that things happen, but I sort of thought, there's even, even though you're giving them a safe avenue to do that there is still some hesitation." 4. Interviews with senior hospital leadership (participant 250) |  |
|  | Individual Identification with Organization | Identification with the organisation was an important reason for which peer messengers decided to take on a formal role in Ethos. There was also identification with the organisation (and its values-see Compatibility) among executive leadership, and conflicting views by these interviewees about whether some staff, particularly medical, identified with the organisation. | moderate | "And that is something that relates to why I've returned three times because there's some connection and a sense of belonging and a sense of identity with our Catholic hospital. So I feel comfortable and that sort of aligns with my own values. So, I would hope that that benefit of being a mission led and values-based hospital means that our personal interactions, reflect those things... the thought that crossed my mind was, to what extent to the medical staff see themselves as being part of the organization, part of the need for the survey." 4. Interviews with senior hospital leadership (participant 180)  "I do know that a lot of the, the medical staff or VMOs [visiting medical officers] that we don't, that aren’t our employees, are very challenged by that, very, very challenged by that but, but John sent out some very good communique that basically said, this is a time for you to reflect. You know, it's not a punitive, it's not going on your record, it's not, you know it's just a reflection, unless it's repeated behaviour and then it will go down a formal. So it's sort of an opportunity for them to reflect." 4. Interviews with senior hospital leadership (participant 250) |  |
|  | Other Personal Attributes | n/a | n/a |  |  |
| Process | Planning | Only one source explicitly dealt with the planning for implementation and outlined at a high level how this would occur within each facility, in collaboration with the wider organisation, with a lead time of 4-6 months and 4-6 months for implementation. The quality of this planning was not commented on either way by other sources, except with specific reference to other constructs (e.g., Available Resources). | minimal | "It is recommended that the implementation process is preceded by an organisational readiness assessment to insure the following are in place to adequately support the implementation of the program: 1. Leadership commitment, 2. Processes and tools for implementation, 3. Supporting resources such as communication and training infrastructure." 1. Program information and implementation plan |  |
|  | Engaging | n/a | n/a |  |  |
|  | 1. Opinion Leaders | n/a | n/a |  |  |
|  | 2. Formally Appointed Internal Implementation Leaders | There was limited information on how formally appointed implementation leaders became engaged with Ethos and given the structure of the organisation (healthcare organisation group and the hospitals that are a part of it) this leadership occurred at both a hospital/facility level and across the group, with some integration. Someone from the chief executive of each hospital was an Ethos sponsor and part of the Group steering committee, suggesting investment of senior leadership at site. In interviews, there were some comments as to the quality of the Ethos coordinators within hospitals, and changes to the role/ | minimal | "The Governance of the Ethos program is provided at facility and Group level. An Ethos Steering Committee has been established and is chaired by [name of Executive from across the group]. Membership of the Steering Committee comprises [names of two other Executives from across the group] and the Chief Executives of each facility where the Ethos program has been implemented." 1. Program information and implementation plan  "So, I think it's great that we've got dedicated staff who are helping to lead and manage the program." 4. Interviews with senior hospital leadership (participant 180) |  |
|  | 3. Champions | Ethos peer messengers have been categorised as champions, because they volunteer to take on a challenging and visible role in the program. Most of the data on how champions became engaged comes from the survey conducted with this group, in addition to interviews. Many respondents had altruistic motivations in taking part in Ethos, believing in its goals and identifying strongly with wanting to improve their organisation. In regards to engagement, a number had been actively sought out by their supervisors for their exceptional professional standing within their hospitals. Some also viewed professional development opportunities from taking on their role. Many participants reported administrative burdens in acting in their role and indicated it was challenging to fit it in with their other work; additionally, there were challenges related to the inherently interpersonal elements of providing Feedback for Reflection. However, {MOST???) were still acting in their role, only a few had moved on. Many found there were rewarding aspects to the role in providing their coworkers with "lightbulb" moment. Other sources suggested variable quality in those who performed this role. | high | "...the messengers sometimes I feel like we're just getting people to do a dirty work. It’s not an easy job. And, that you know, I’ve already seen some backfires on messengers and not very nice for them. And they're not being paid for what they do, whereas I’m being paid to take the heat." 4. Interviews with senior hospital leadership (participant 220)  "We've been very fortunate that our doctors put their hands up, straight away. And we did that, the training session with them, which I’m sure you heard of. And we've got another staff training next week so they're messengers, there's a core group but we've realized there's some gaps, but it can't be the one person delivering all the messages." 4. Interviews with senior hospital leadership (participant 230)  "I am passionate about staff and patient safety." 5. Survey of Ethos peer messengers  "Encourages transparency, helps to bridge the gap when behaviours risk patient and team safety in a non-threatening manner." 5. Survey of Ethos peer messengers  "I thought it would be a great chance to learn and help develop how to deal with situations that arise in the work place." 5. Survey of Ethos peer messengers  "I was asked to do this job and after the training session I thought I was up to the task." 5. Survey of Ethos peer messengers  "I was approached to be a messenger - I was probably volunteered by my manager as a representative from my department. It was also an opportunity for me to do something outside my comfort zone." 5. Survey of Ethos peer messengers  "When the ETHOS was first getting rolled out, there was meetings, so we had a general manager of the hospital. I had a meeting with her, and she said would you like to be a part of it, with rolling out? I said why not, it’s a good learning opportunity, why not be part of it, learn from it and see where it goes?" 6. Interviews with middle managers | enabler |
|  | 4. External Change Agents | n/a | n/a |  |  |
|  | 5. Key Stakeholders (Staff) | As a whole-of-hospital program, all staff are expected to enact this innovation. Staff are also the primary target of it, rather than the patients. In addition to training and resources (coded elsewhere), participants reported poor engagement and participation for themselves or particular stakeholder groups within the hospital (e.g., VMOs, anaesthetists) due to their role or past experiences of interpersonal issues at their hospital. Introductions to Ethos provided to all staff were also sometimes planned for inconvenient times. Some mentioned that a lot of this initial promotion of Ethos and encouragement to use its reporting tool had subsided. Training, intended to be offered to everyone, was not always delivered. There were conflicting perspectives on how aware of the program hospital staff were, suggesting limitations in engagement strategies and certainly in their outcome. Attempts to engage specific stakeholders (VMOs) was mentioned by one interviewee, but this was conducted by individual initiative. | moderate | "I have had a previous experience with bullying by a manager. I left the job because of it. Now that person is on the poster for Ethos. I am less likely to engage with the program because of that." 3. Baseline survey of all staff (Hospital 1)  "It would be great if there was better doctor engagement." 5. Survey of Ethos peer messengers  "Inclusion of more representative professionals i.e., surgeons, cardiologists, anaesthetists, intensivists, ED, geriatrician to advocate for staff." 5. Survey of Ethos peer messengers  "Whereas before when it launched there was a lot of publicity about it and a lot of encouragement to put in positive and negative reports." 6. Interviews with middle managers |  |
|  | 6. Innovation Participants (Patients) | n/a | n/a |  |  |
|  | Executing | There were a few reports across sources about instances where Ethos as implemented did not meet the intentions and some of the specific plans of the program, with examples of feedback not being delivered in a timely fashion, staff not being provided with skills to address unprofessional behaviour in situ, too much focus on the negative reports, and Ethos messengers having to deliver messages that should not have been passed on. Among senior leadership, there were also challenges reported in adhering to the escalation processes for reports, and accidentally using information from Ethos in a formal HR review. | minimal | "My manager made a positive report about my work to Ethos and was promised that I would receive a feedback session from Ethos. This was earlier in the year and I have had no feedback or contact from the Ethos team. If the Ethos team want to provide a stream for positive feedback (as well as negative), there needs to be good follow-up processes to help staff feel valued for their contributions." 3. Baseline survey of all staff (Hospital 1)  "Oh look, I just think follow the program, the ETHOS program, that the Group have run out. I think they brought it out really well. I think they had a really good system in place for training, for how the triage team worked in terms of filtering the messages that had been received, and working out whether or not they needed to be delivered, and how they needed to be delivered, and who [they] needed to deliver them to." 6. Interviews with middle managers |  |
|  | Reflecting and Evaluating | There was a clear commitment to reflection and evaluation, including by formal review and less formal debriefing sessions with program champions. However, there were some reports that the data being captured could be more meaningful. An independent review was commissioned to understand how the program was working in the two sites that were first to implement; this led to recommended revisions, some of which were enacted. Data collected for the research evaluation project, particularly the survey of Ethos peer messengers, also sought feedback on progress, challenges and things that could be improved. Some interviewees indicated data sources they drew upon to understand progress of the program (e.g., levels of awareness among staff, utilisation rates for the reporting tool). Responses suggested a desire for more meaningful data, and for feedback to be collected from recipients of messages and Ethos peer messengers. In interviews, considerations of qualitative and quantitative feedback were more concerned with demonstrating program effectiveness, rather than implementation, with scepticism that data would show an effect. However, senior managers also mentioned instances of informal information-gathering around whether (adoption) and how (fidelity) Ethos was being used, as well penetration and acceptability. | high | "This will continue to be tailored to the site, with cyclical feedback coming from regular peer mentor sessions, triage team reviews and analysis of staff engagement and satisfaction." 1. Program information and implementation plan  "In March 2019, the [hospital group] commissioned an independent review of the Ethos program to identify which elements of the program are working well, and which elements could be improved and how. The scope of the review extended to Ethos at both [hospital name] and [other hospital name], to consider whether the program is fair to all parties, including the pros and cons of anonymous reporting... Ethos will continue to be evaluated as part of a longitudinal investigation in partnership with the [research group], with whom [the hospital group] share a $1.2m NHMRC partnership grant to evaluate the program." 2. Independent review of Ethos program in two hospitals  "...the improvement work and Ethos and really improvement engagement, and perhaps reduction in that behaviour that is inappropriate... it’s a really difficult experiment, cos you've got two things, they're impacting the same thing. How do you measure which one is doing what? I don’t know, that’s the challenge for you I guess." 4. Interviews with senior hospital leadership (participant 140)  "...what I will be very interested in seeing at the end, you know how many Ethos reports are made, how many of them are unactionable. And how many of those are getting to the, the end of the behaviour that we're actually trying to address because, you know, is it being used, I guess in a way that isn't helpful. The other thing I'll be very interested in obviously, the outcomes. So, what are the experiences of people who have been delivered an Ethos message and where did it, where did it feel right and where didn’t it feel right. You know, I guess my other concern is how skilled our, our triage team is." 4. Interviews with senior hospital leadership (participant 170)  "Regular training/workshops particularly because it can be sometime between messages. Feedback is possible on how your message was delivered." 5. Survey of Ethos peer messengers  "I think it's getting better. So we have about 85 per cent of the people aware of Ethos and what it is, but we're only 12 months in and I don't think that we'll see change for another four years. So my sort of model was for the first year get people to know what is Ethos and the second one is actually talk about assertiveness. So training, speaking up at the time, so then we'd see less reports coming through." 6. Interviews with middle managers | enabler |
