# Supplementary material for: Retrospective analysis of factors influencing the implementation of a program to address unprofessional behaviour and improve culture in Australian hospitals
Source: BMC Health Serv Res. 2023 Jun 7;23:584. doi: 10.1186/s12913-023-09614-1 (PMC10244846; doi:10.1186/s12913-023-09614-1)
Supplement: Supplementary file 4 — Additional file 4. [file 12913_2023_9614_MOESM4_ESM.docx]

| **ERIC Strategy** | **Operationalisation by program implementers** | **CFIR constructs matched to strategy*** | **Description of barrier(s) in context** | **Alignment between strategy and barrier in context** | **Overall assessment of alignment** |
| --- | --- | --- | --- | --- | --- |
| Conduct educational meetings | Educational meetings and training were held with the different stakeholders (line managers, executive, peer messengers, and all other staff). | Design Quality & Packaging | Poor perceptions of the reporting tool and process, scepticism about anonymity, dislike of the lack of “natural justice”, confusion about the inclusion of both positive and negative reports in the one system. | Aligned - educational meetings to introduce different stakeholders to the program. | Partially aligned |
|  |  | Goals & Feedback | Broad goals but lack of clarity about what the program would achieve, perpetuated by the dearth of feedback at both an individual and organisational level on usage of Ethos. | Partially aligned - meetings were held, but not on an ongoing basis and not using content from the reporting system. |  |
|  |  | **Access to Knowledge & Information** | Training not accessible to all, visibility of Ethos declined over time and specific information on the reporting process was inadequate. | Not aligned - training not always accessible despite being a whole of hospital intervention, also not sustained |  |
|  |  | **Knowledge & Beliefs about the Intervention** | On balance, beliefs were distrusting, sceptical or measured in their view of the program and how effective it could be. | Aligned - educational meetings to introduce different stakeholders to the program. |  |
| Identify and prepare champions | Ethos peer messengers were program champions with evidence of selection by senior staff and training and support provided to them. | **Learning Climate** | Hospitals were viewed as frequently hostile and punitive when issues were raised. Spurious focus on quality improvement. | Aligned – Peer messengers wanted to contribute to quality, safety and workplace culture, and were professional in delivery of feedback that encouraged self-reflection, not blame. | Aligned |
|  |  | Leadership Engagement | Staff perceived leaders as not accountable with a poor track record of addressing unprofessional behaviours. Senior leaders were supportive of Ethos but not core implementers and not always knowledgeable on the program. | Aligned – Ethos peer messengers were frequently supported, and even selected by, hospital leadership. |  |
|  |  | Access to Knowledge & Information | Training not accessible to all, visibility of Ethos declined over time and specific information on the reporting process was inadequate. | Aligned – Many Ethos peer messengers took it upon themselves to explain and encourage use of the program. |  |
| Facilitation | Not identified. |  |  |  | Not identified. |
| Conduct local consensus | Not identified. |  |  |  | Not identified. |
| Develop educational materials | An Ethos procedure document, as well as promotional materials, were developed for staff and went through several versions. | Design Quality & Packaging | Poor perceptions of the reporting tool and process, scepticism about anonymity, dislike of the lack of “natural justice”, confusion about the inclusion of both positive and negative reports in the one system. | Aligned – materials developed and subject to multiple iterations in an attempt to clarify program processes. | Aligned |
|  |  | **Access to Knowledge & Information** | Training not accessible to all, visibility of Ethos declined over time and specific information on the reporting process was inadequate. | Aligned - a range of materials were developed from detailed program descriptions to promotional screensavers, and went through multiple iterations as required. |  |
|  |  | Knowledge & Beliefs about the Intervention | On balance, beliefs were distrusting, sceptical or measured in their view of the program and how effective it could be. | Aligned – materials produced over time attempted to correct misconceptions and knowledge gaps. |  |
| Organise clinician implementation team meetings | Not identified. |  |  |  | Not identified. |
| Create a learning collaborative | An inter-hospital Ethos working group was established to share challenges and issues of those implementing the program. | Networks & Communication | Poor communication vertically and horizontally, despite procedures for consultation. Networks characterised by lack of transparency, hierarchy, and tribalism. | Not aligned – Issues occurred within each hospital, while collaborative occurred at the group level. | Not aligned |
|  |  | Access to Knowledge & Information | Training not accessible to all, visibility of Ethos declined over time and specific information on the reporting process was inadequate. | Partially aligned - training not ongoing, visibility of educational materials declined, so collaborative learnings did not trickle down effectively. |  |
| Inform local opinion leaders | Not identified. |  |  |  | Not identified. |
| Capture and share local knowledge | Not identified. |  |  |  | Not identified. |
| Develop a formal implementation blueprint | The program information and implementation plan laid out the aims/purposes of Ethos, scope (all staff), and included a high-level timeline for implementation in each hospital. | Goals & Feedback | Broad goals but lack of clarity about what the program would achieve, perpetuated by the dearth of feedback at both an individual and organisational level on usage of Ethos. | Partially aligned – Blueprint included only broad goals and strategies. Performance measures and plans for refining the plan were not specified. | Partially aligned |
|  |  | Leadership Engagement | Staff perceived leaders as not accountable with a poor track record of addressing unprofessional behaviours. Senior leaders were supportive of Ethos but not core implementers and not always knowledgeable on the program. | Aligned - Commitment of leaders at a group level and strategies for engaging leadership at a hospital level clearly specified. |  |
| Promote adaptability | Not identified. |  |  |  | Not identified. |
| Build a coalition | Not identified. |  |  |  | Not identified. |
| Audit and provide feedback | Not identified. |  |  |  | Not identified. |
| Promote network weaving | The program information and implementation plan mentioned the plan to continue “to engage external stakeholders". | Networks & Communication | Poor communication vertically and horizontally, despite procedures for consultation. Networks characterised by lack of transparency, hierarchy, and tribalism. | Not aligned – poor communication, tribalism, occurred within each hospital, while networking was done externally or at a group level. | Not aligned |
| Distribute educational materials | Program materials promoting Ethos including posters and screensavers distributed. FAQ and web portal created. Multiple versions created over time. | Access to Knowledge & Information | Training not accessible to all, visibility of Ethos declined over time and specific information on the reporting process was inadequate. | Partially aligned – materials distributed initially, but not effectively over time. | Partially aligned |
| Recruit, designate and train for leadership | Both line managers and senior hospital leaders undertook specialised training to support their involvement and leadership of Ethos, and some had designated roles. | Learning Climate | Hospitals were viewed as frequently hostile and punitive when issues were raised. Spurious focus on quality improvement. | Partially aligned - multiple roles trained up to support implementation of program, but unclear that this would affect the largely hospital learning climate. | Partially aligned |
|  |  | Leadership Engagement | Staff perceived leaders as not accountable with a poor track record of addressing unprofessional behaviours. Senior leaders were supportive of Ethos but not core implementers and not always knowledgeable on the program. | Partially aligned – strategy addressed engagement with the program more than leadership accountability issues. |  |
| Conduct educational outreach visits | Not identified. |  |  |  | Not identified. |
| Conduct local needs assessment | Not identified. |  |  |  | Not identified. |
| Use an implementation adviser | Not identified. |  |  |  | Not identified. |
| Assess for readiness and identify barriers and facilitators | Assessment of readiness for implementation was planned for each site 4-6 months prior to implementation. | Knowledge & Beliefs about the Intervention | On balance beliefs were distrusting, sceptical or measured in their view of the program and how effective it could be. | Partially aligned – limited detail on how assessment conducted, and lack of evidence to suggest it adequately evaluated knowledge and beliefs of staff prior to implementation. | Partially aligned |
| Alter incentive/allowance structures | Not identified. |  |  |  | Not identified. |
| Identify early adopters | The independent review examined the process of adoption at two sites first to implement Ethos and learned from their experience. | Knowledge & Beliefs about the Intervention | On balance beliefs were distrusting, sceptical or measured in their view of the program and how effective it could be. | Aligned – from analysis of early implementing sites, identified a range of misconceptions about the program and how it worked, recommended improvements. | Aligned |
| Facilitate relay of clinical data to providers | Not identified. |  |  |  | Not identified. |
| Use advisory boards and workgroups | Ethos Action Plan Working Group created with both internal Ethos program leads from each hospital and some external academics. In a series of formal meetings, oversaw refinements to the program following Internal Review. | No individual barrier with Level 1 or 2 endorsement. | N/A | N/A | Not applicable |
| Conduct ongoing training | Not identified. |  |  |  | Not identified. |
| Involve executive boards | The program had the full support of the group executive, and at a hospital level, a member of the executive was designated the Ethos sponsor. Engagement sessions were run with hospital executive and senior leaders so they could support the program. | Leadership Engagement | Staff perceived leaders as not accountable with a poor track record of addressing unprofessional behaviours. Senior leaders were supportive of Ethos but not core implementers and not always knowledgeable on the program. | Partially aligned – addressed program engagement but not accountability issues with leadership. | Partially aligned |
| Provide ongoing consultation | Not identified. |  |  |  | Not identified. |
| Develop and implement tools for quality monitoring | Not identified. |  |  |  | Not identified. |
| Obtain and use patients/consumers and family feedback | Not applicable. |  |  |  | Not applicable |
| Model and simulate change | Not identified. |  |  |  | Not identified. |
| Involve patients/consumers and family members | Not applicable. |  |  |  | Not applicable |
| Obtain formal commitments | The program information and implementation plan specified the full commitment of the group’s board and outlined various roles and responsibilities of key leaders and Ethos sponsors. Commitments were also made related to the evaluation of the Ethos program. | Leadership Engagement | Staff perceived leaders as not accountable with a poor track record of addressing unprofessional behaviours. Senior leaders were supportive of Ethos but not core implementers and not always knowledgeable on the program. | Partially aligned – numerous key partners were not in a leadership relationship. Commitment from leadership explicit at a group but not hospital level. | Partially aligned |
| Purposely re-examine the implementation | The independent review and the researcher-led evaluation of the program implementation were conducted. A working group was established, and an action plan put into place following this to ensure recommendation revisions were adopted. | Design Quality & Packaging | Poor perceptions of the reporting tool and process, scepticism about anonymity, dislike of the lack of “natural justice”, confusion about the inclusion of both positive and negative reports in the one system. | Aligned – evidence of materials and strategies being revised in light of new information and recommendations. | Aligned |
| Increase demand | Not applicable. |  |  |  | Not applicable |
| Provide local technical assistance | Not identified. |  |  |  | Not identified. |
