# Supplementary material for: Retrospective analysis of factors influencing the implementation of a program to address unprofessional behaviour and improve culture in Australian hospitals
Source: BMC Health Serv Res. 2023 Jun 7;23:584. doi: 10.1186/s12913-023-09614-1 (PMC10244846; doi:10.1186/s12913-023-09614-1)
Supplement: Supplementary file 5 — Additional file 5. [file 12913_2023_9614_MOESM5_ESM.docx]

**Example of how to use theory in the implementation of a professional accountability program.**

| **Assess readiness for implementation** | **Barrier identified** | **Implementation strategy recommended** | **Operationalisation of strategy** |
| --- | --- | --- | --- |
| Interviews conducted with cross section of staff (middle and senior management, staff from clinical/non-clinical, varying seniority and degrees of “power”) guided by the CFIR. | *Leadership engagement* poor, with a perceived lack of commitment to addressing unprofessional behaviour. | Conduct local consensus discussions | Minimum 6 months prior to implementation, hospital leadership conduct multiple public forums ensuring coverage of all staff groups. Transparently discuss increased awareness of, and commitment to addressing, unprofessional behaviour, the wider hospital strategy for addressing serious behaviour, and seek feedback on the program. |
